# A late Middle Pleistocene Middle Stone Age sequence identified at Wadi Lazalim in southern Tunisia

**DOI:** 10.1038/s41598-022-07816-x

**Published:** 2022-03-18

**Authors:** Emanuele Cancellieri, Hedi Bel Hadj Brahim, Jaafar Ben Nasr, Tarek Ben Fraj, Ridha Boussoffara, Martina Di Matteo, Norbert Mercier, Marwa Marnaoui, Andrea Monaco, Maïlys Richard, Guido S. Mariani, Olivier Scancarello, Andrea Zerboni, Savino di Lernia

**Affiliations:** 1 Dipartimento di Scienze dell’Antichità, Sapienza University of Rome, Rome, Italy; 2 Artisanat du Sahara, Douz, Tunisia; 3 Faculté des Lettres et des Sciences Humaines, Université de Kairouan, Kairouan, Tunisia; 4 Faculté des Lettres et des Sciences Humaines, Université de Sousse, Sousse, Tunisia; 5 Laboratoire de Cartographie Géomorphologique des Milieux, des Environnements et des Dynamiques (CGMED), Université de Tunis, Tunis, Tunisia; 6 Institut National du Patrimoine, Tunis, Tunisia; 7 Archéosciences-Bordeaux, UMR 6034 CNRS-Université Bordeaux Montaigne, Pessac, France; 8 Centro Nacional de Investigación sobre la Evolución Humana (CENIEH), Burgos, Spain; 9 Dipartimento di Scienze Chimiche e Geologiche, Università degli Studi di Cagliari, Cagliari, Italy; 10 Dipartimento di Scienze della Terra “A. Desio”, Università degli Studi di Milano, Milano, Italy; 11 School of Geography, Archaeology and Environmental Studies, University of the Witwatersrand, Johannesburg, South Africa

**Keywords:** Archaeology, Geomorphology

## Abstract

The late Middle Pleistocene, starting at around 300 ka, witnessed large-scale biological and cultural dynamics in hominin evolution across Africa including the onset of the Middle Stone Age that is closely associated with the evolution of our species—*Homo sapiens*. However, archaeological and geochronological data of its earliest appearance are scarce. Here we report on the late Middle Pleistocene sequence of Wadi Lazalim, in the Sahara of Southern Tunisia, which has yielded evidence for human occupations bracketed between ca. 300–130 ka. Wadi Lazalim contributes valuable information on the spread of early MSA technocomplexes across North Africa, that likely were an expression of large-scale diffusion processes.

## Introduction

The emergence of the Middle Stone Age (MSA) in North Africa has been traditionally considered the result of *H. sapiens* dispersals triggered by late Middle Pleistocene ecosystem fragmentation from areas of endemism in East Africa, where early *H. sapiens* fossils dated to ca. 200 ka^1^ have been found; Sangoan and Lupemban techno-complexes would have represented the archaeological signature for these first expansions^2^. This framework has been questioned in light of African Multiregionalism concepts building upon a series of independent data^3^, including the dating of MSA assemblages associated to *H. sapiens* fossils to ca. 315 ka at Jebel Irhoud^4,5^, as well as attempts for a more regional interpretation of technological sequences and variation^6,7^.

Late Middle Pleistocene human biogeography of North Africa is directly linked to the Sahara, which has long been identified as a driver for biological diversification and population separation^8,9^. However, while being recognized as a critical area for human evolution, our current understanding of the Saharan biogeographic role remains speculative because its archaeological, geochronological, and paleoanthropological data are too scarce and poorly distributed to resolve the relationships between its northern and southern regions at this critical time period.

Here, we report results from investigations at Wadi Lazalim in southern Tunisia, at the northern edge of the Sahara. The area preserves an open-air sedimentary sequence of mostly late Middle Pleistocene age—dating from at least Marine Isotopic Stage (MIS) 8 to the transition from the MIS6 to MIS5 interglacial. The archaeological evidence from lithic assemblages in sub-primary deposition comprises well-recognizable technological elements for which infrared luminescence data provide some chronometric insight revealing fresh information on early MSA human occupation of the northern Sahara and contributing new elements for the discussion about the spread of MSA techno-complexes from sub-Saharan Africa into North Africa in the late Middle Pleistocene.

## Results

**Figure 1:**
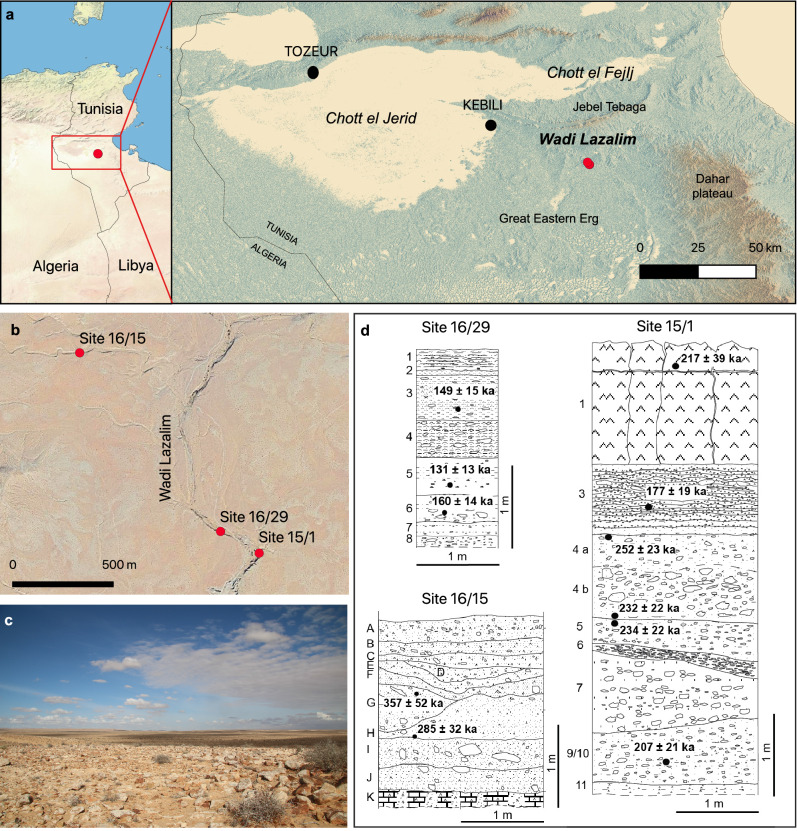
Geographic and stratigraphic setting of the MSA sites at Wadi Lazalim (maps created using QGIS 3.20.2 https://qgis.org/it/site/). **a**: localization and main geographic features of the research area (base maps: left, made with Natural Earth; right, made with SRTM 1 ArcSec); **b**: satellite view of the area encompassing the excavated sites (Google Earth); c: view of Wadi Lazalim from the heights north of site 16/15 looking eastwards; d: drawing of the excavated stratigraphic sections at Site 16/29 (South profile), Site 16/15 (South profile) and Site 15/1 (East profile) with indication of results from the IRSL dating (description of the sedimentary units in Supplementary Information); numbers and letters to the left of each section indicate layer names.

Wadi Lazalim is in the region of Kebili, in southern Tunisia (Fig. 1a,b). Its watershed (51.4 km^2^) drains part of the Jebel Daouaia and the hills along its west side. Local geological series outcropping include Lower Campanian clay and marl with thin limestone strata, capped by Upper Campanian limestone containing chert nodules^10,11^. Quaternary formations and landforms (Fig. SI 1) include a first erosion glacis confined to the foothills formed by a consolidated deposit about 2 m thick and sealed by a gypsum duricrust 0.5 to 1 m thick. A second erosion glacis 3 to 4 m thick is formed by friable to consolidated deposits lying on Lower Campanian gypsum clays and marls and covered by a gypsum duricrust 0.7 to 1 m thick. A piedmont alluvium, developed following the partial destruction of the glacis, is constituted by moderately cemented deposits sealed by a 10 to 20 cm thick gypsum crust. A 1.5 m thick terrace of coarse heterogeneous deposits is buried below the two glacis.

Three test trenches were excavated along the fluvial cuts of the Wadi Lazalim and its tributaries at sites 16/15 (N 33,520,633 E 9,416,977; trench size: 2 m wide, 2.1 m deep), 15/1 (N 33,509,899 E 9,426,784; trench size: 2 m wide, 5.2 m deep), 16/29 (N 33,511,043 E 33,511,043; trench size: 1 m wide, 2.4 m deep). The sedimentary sequences were exposed by the development of river dynamics activating longitudinal and lateral gullies from the structural reliefs. Stratigraphic sequences (Fig. 1d) consist of successive massive and consolidated layers, dominated by coarse deposits of sub-angular blocks and gravels in a sandy to silty matrix, eventually interlayered by lenses of sand and silt rich in gypsum and limestone clasts (see description of the sedimentary units provided in SI text and Fig. SI 2). The progressive gypsum enrichment along the sequences—controlled by evapotranspiration – suggests the transition towards progressively more arid environmental conditions, whose apogee corresponds to the formation of the gypsum duricrusts at the topsoil. Sedimentary facies enables the interpretation of the sequences as alluvial/colluvial bodies accumulated in semi-arid environmental conditions by surface runoff, alternating erosional (truncation) and depositional events. Occasionally, deactivation of surface processes was switched on/off by alternating interglacial/interstadial and glacial/stadial phases, up to the general interruption of sedimentation (and likely a major erosive event) in the Last Glacial phase. A trend towards increasing aridity and diminished length of interglacial conditions and slowing of surface processes is recorded in many areas of the Sahara^12,13^ and several activations of fluvial systems are recorded over North Africa^13–15^, possibly correlating to enhanced fluvial dynamics at the margin of Tunisian Chotts^16^.

Infrared Stimulated Luminescence dating (IRSL) has been applied to date the three sites; pIRIR_290_ ages obtained on single feldspar grains indicate a deposition time spanning the second half of the Middle Pleistocene to the threshold of the Late Pleistocene. Site 16/15 layer G returned an age of 357 ± 52 ka, overlapping with the one obtained for the underlying layer H (285 ± 32 ka) and suggesting a deposition time likely before MIS 8. Site 15/1 layer 1 was dated to 217 ± 39 ka whereas layer 3 yielded an age of 177 ± 19 ka, indicating a deposit of these layers either during the second part of MIS 7 or the onset of MIS 6. In the same site, the four ages obtained from layers 4a (252 ± 23 ka), 4b (232 ± 22 ka), 5 (234 ± 22 ka) and 9/10 (207 ± 21 ka), are potentially slightly older and could be included in MIS 7–8. Site 16/29 provided the youngest age results: 149 ± 15 ka for layer 3, 131 ± 13 ka for layer 5 and 160 ± 14 ka for layer 6; they all fall between MIS 6 and the onset of MIS 5. Consequently and despite the low resolution of these data, all these deposits likely cover a long time range from MIS 8 up to MIS 6, or even MIS 5.

The archaeological record here consists of 1135 lithic artefacts (Table SI 4). Flint is the only raw material. The most common type is a brownish variety, rather coarse grained, which is strictly local and comes from a nearby outcrop (< 1 km) of upper Cretaceous age, where it is present in the form of large boulders and nodules (Fig. SI 7). Far less abundant are fine grained, vitreous, multicolor flints, whose incidence accounts for around 8% globally, the source of some of which have been locally identified in a range of about 10 km around the sites (Fig. SI 7).

The lithic artefacts show extensive post-depositional alterations. The most widespread can be ascribed to chemical weathering, resulting in patination, pits, striation, exfoliation. Transport mechanical alteration, in a tribological sense^17^, producing edges and ridges damage and rounding is also recognizable, though less frequent.

Preliminary taphonomic investigations are based on a sample of artefacts (n = 241) from the three excavated sequences. Three groups of artefacts distinguished on the basis of macroscopic observation of overall surface alteration can be defined (Fig. SI 8): Group 1 has extensive chemical weathering with surface alteration; mechanical action not fully assessable; Group 2 has diffuse patination without appreciable surface corrosion; mechanical action, when present, is generally visible and assessable and Group 3 has moderate to no presence of chemical weathering also resulting in light to absent patination; edges and ridges are lightly affected by mechanical alterations.

The co-occurrence of artefacts with different degrees of alteration (Fig. SI 8) first suggests that the composition of the archaeological assemblages derives from natural depositional processes which displaced artefacts already exposed to weathering before their eventual burial. Nevertheless, the effects of weathering on lithic artefacts are not easy to predict. They vary by both artefact mineralogy and environmental conditions, including the geochemical properties of the sedimentary deposits^18–21^, the combination of which can result in highly selective and differentiated modes of attack during the taphonomic processes. The basic assumption that fine grained flint resist weathering better than coarse grained one^20^, and the recognition that most of the strongly weathered artefacts (Group 1) are made on medium-coarse grained flint, whereas fine and very fine grained flint artefacts show a good preservation of edges and ridges, lighter surface alteration and little to no patination (Groups 2 and 3), significantly narrows down the seeming heterogeneity and suggests the likelihood that the observed differential alteration largely depended on raw material type.

The above defined groups, first defined by macroscopic observation of surface alteration and grossly correlating with raw material quality, finds further consistency in overall metric features (Tab. SI 5, Fig. SI 9), indicating that the artefacts on fine grained flint are largely and significantly smaller than the artefacts on medium/coarse grained flint. Further, a statistically significant difference among the three groups, as determined by one-way ANOVA test, can be advanced when artefacts’ length (F (2.238) = 3.88, *p* = 0.002) and width (F (2.238) = 3.118, *p* = 0.046) are considered. While technological, economic and land use strategies can be all invoked to be responsible for overall assemblage metric attributes, it can be also inferred that these records depend on an inferably varied granulometric composition of the natural deposits where the raw blocks of raw material were collected.

Some indications about the collecting environments come from the type of cortex. It can be inferred that raw materials were largely procured at primary outcrops or secondary deposits, including fluvial ones, close to their primary geological sources. This is indicated by the preponderance of pieces, among the cortical ones, with a slightly weathered natural cortex mainly showing a nodular morphology (Fig. SI 10). Remarkably, the few fine grained flint cortical artefacts only show rounded neocortical surfaces, suggesting that the procurement of at least part of the highest quality raw materials targeted flint pebbles available at secondary contexts like fluvial deposits and riverbeds, also locally.

The overall technological composition of the lithic assemblages (Table SI 4, Fig. SI 11) mainly includes ordinary flakes, which are the most represented at all sites (16/15 = 77.9%; 15/1 = 59.1%; 16/29 = 59.7%). Unretouched predetermined end-products globally show an incidence at 16/15 = 8.9%; at 15/1 = 8.6%; at 16/29 = 9.7%. Retouched/shaped blanks are numerous and account for 16/15 = 11.3%, 15/1 = 18.9%, 16/29 = 21%. Cores are variably represented (16/15 = 1.1%; 15/1 = 6.5%; 16/29 = 3.2%).

**Figure 2:**
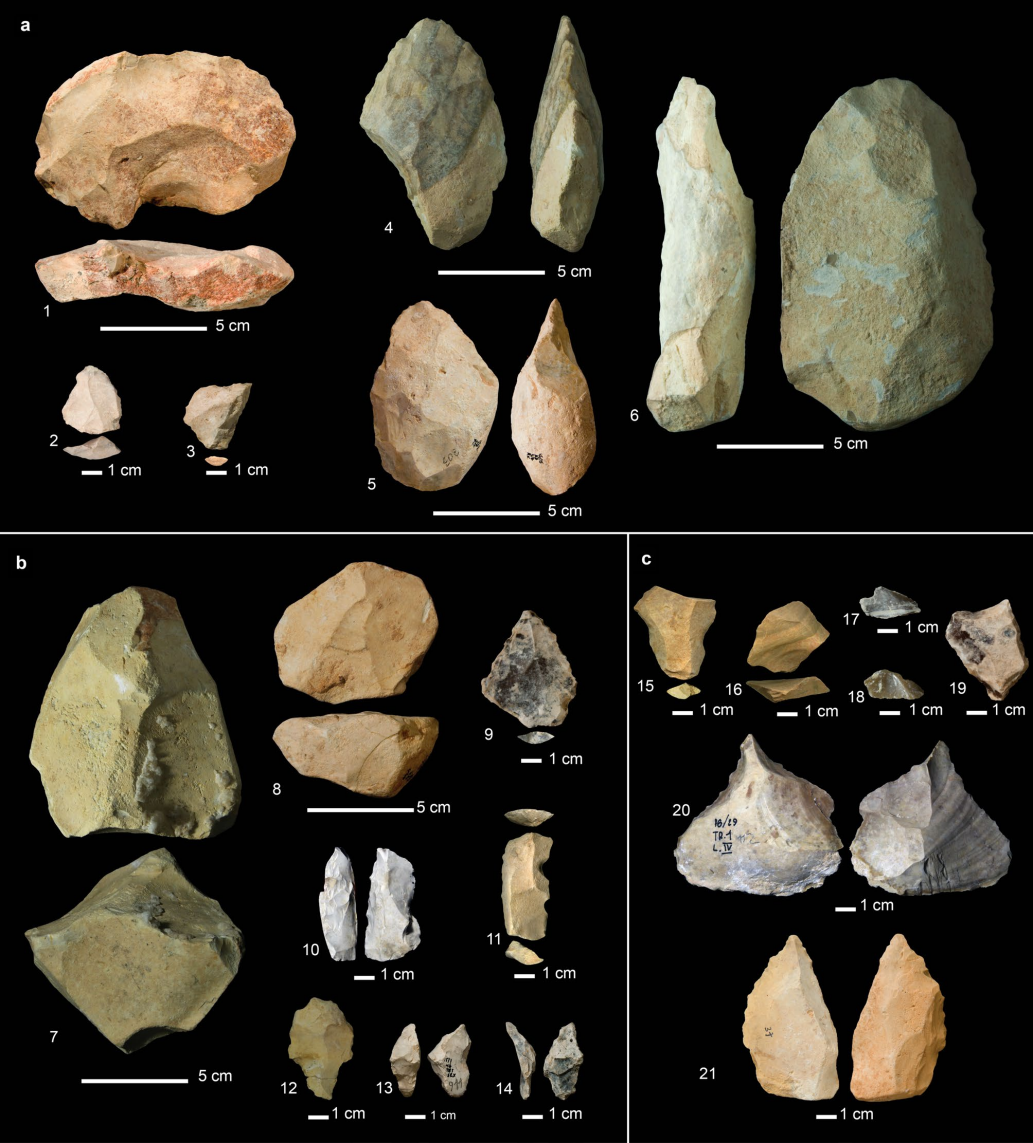
Archaeological materials discussed in the text. a, Site 16/15: 1, Levallois core (Layer I); 2, Levallois flake (Layer J); 3, convergent scraper (Layer I); 4–5, bifacial knives (layer H); 6, core-axe (layer H). b, Site 15/1: 7, blade core (Layer 7); 8, Levallois point core (layer 4a); 9, Levallois point (layer 4b); 10, side-scraper (Layer 4b); 11, end-scraper (Layer 5); 12, tanged tool (layer 5); 13, backed piece (layer 4b); 14, backed piece (layer 4a). c, Site 16/29: 15, notch on a Levallois flake (Layer 6); 16, discoid flake (Layer 6); 17–18, flakes from bifacial reduction (Layer 3 and 4 respectively); 19, tanged tool (layer 7); 20, beck (Layer 4); 21, point (outside excavated trench, same sedimentary sequence).

At site 16/15 (720 artifacts, Table SI 4) technological features indicate an extensive use of the Levallois method, which is testified by end-products (e.g. Fig. 2 n. 2) and one centripetal recurrent Levallois core from layer I (Fig. 2 n. 1, length = 117 mm, width = 73 mm, thickness = 28 mm, Tab SI 6). Altogether, the Levallois blanks mainly show centripetal and unipolar dorsal scars (Tab. SI 7).

Levallois blanks, both retouched and not retouched, are of relatively small size across the entire sequence and never exceed 100 mm; this threshold also applies to fragmented specimens, which partly rules out the possibility that observations based on whole pieces only are biased. There is an exception for one case from the topmost layer A. Global site’s means calculated on 41 whole Levallois blanks are: length = 58.1 mm, width = 43.3 mm, thickness = 11.4 mm (see Tab. SI 9 for details on individual layers).

Laminar blanks are common, they have mostly plain platforms and prevalently unipolar dorsal scars (Table SI 10, Tab. SI 11). Overall metric assessment based on whole laminar blanks, both retouched and unretouched, indicates they are of generally small size not exceeding 82 mm (which also applies to fragments). Metric mean values calculated on 9 whole blanks across layers of 16/15 site are: length = 53.0 mm, width = 25.7, thickness = 11.5 mm (Tab. SI 12).

Other reduction methods are more rarely recognized, but they anyhow suggest variably organized technological systems. Among these are the Discoid and the Kombewa, the latter term used here to designate simple core-on-flake technologies. End-products, but no cores, have been identified only in the lowermost layers I and J. The few complete specimens recovered have maximum dimensions of 69 mm and 54 mm respectively (Tab. SI 13).

The retouched flake/blade tool inventory (Tab SI 4, Fig. SI 12) includes scrapers, among which some are convergent with a sharply pointed *déjeté* end (Fig. 2 n. 3), denticulates, notches, end-scrapers, truncations and becks.

Flake/blade tools do not exceed 83 mm (fragments also), except for layer I where these can be up to 154 mm. Metric mean values calculated on 47 whole blanks across layers of 16/15 site are: length = 56.2 mm, width = 43.9 mm, thickness = 12.5 mm (see Tab SI 14 for details on individual layers).

Common flakes constitute most of the retouched blanks (Tab. SI 15). A considerable part of the tools is made on predetermined blanks such as blades (ca. 7–40%) and Levallois flakes (ca. 14–50%). The latter’s sizes are almost fully encompassed by those of the unretouched ones (Fig. SI 13).

Site 16/15 yielded four bifacial tools. One of these, that was deeply weathered and rounded, comes from the uppermost layers (B-C). Three other ones come from layer H; although patinated, they show little mechanical alteration. One bifacial tool is made on a flake (Fig. 2 n. 4, length = 109 mm, width = 69 mm, thickness = 33 mm), has a thick proximal end and a thick back. The shaping is made by flat retouch. The second (Fig. 2 n. 5, length = 90 mm, width = 59 mm, thickness = 35 mm) is made on a nodule, has a thick cortical back, the base is bifacially shaped. The point is thinned and it was possibly re-sharpened. The third (Fig. 2 n. 6, length = 169 mm, width = 98 mm, thickness = 47 mm) is made on a cortical flake from a nodule. The distal end is blunted and accurately shaped. The rest is left unworked or roughly shaped, with a sinuous profile. According to the definition by Clark and Kleindienst^22^, both first two specimens can be classified as knives, while the latter can be described as a core-axe. Finally, a biface roughout comes from layer J, made on a large thick cortical flake.

At site 15/1 (291 artifacts, Table SI 4) the Levallois method is testified by end-products and by two cores: one is for flakes (layer 9/10, length = 51 mm, width = 47 mm, thickness = 19 mm); the other one is for points (layer 4a, Fig. 2 n. 8, length = 57 mm, width = 63 mm, thickness = 34 mm). Dorsal scars on Levallois flakes suggest unidirectional and bidirectional recurrent mode of production (Tab. SI 7). Levallois flakes are rather small and do not exceed 71 mm (which also applies to fragments). Metric mean values calculated on 10 whole blanks across layers of 15/1 site are: length = 50.1 mm, width = 35 mm, thickness = 10 mm (Tab. SI 9). One Levallois point comes from layer 4b (Fig. 2 n. 9, length = 56 mm, width = 45 mm, thickness = 8 mm).

The presence of laminar blanks is frequent throughout the sequence. Some have facetted platforms and multidirectional dorsal scars that may well indicate a Levallois conception of core reduction. Other specimens show plain platforms and unipolar dorsal scars (Tab. SI 10, Tab. SI 11) and may indicate a different (non-Levallois) laminar conception of core reduction, as testified by the flat striking platform and the unipolar removals visible on a core from layer 7 (Fig. 2 n.7, length = 150 mm, width = 91 mm, thickness = 84 mm). Blade blanks are small and do not exceed 71 mm (which also applies to fragments). Global mean dimensions calculated on 10 whole specimens are length = 52, width = 23 mm, thickness = 9.8 mm (Tab. SI 12), comparable to the Levallois flakes.

The Discoid method was recognized based on end-products and two cores (layers 4b, 5 and 7), which attest to the rather small size of this production (cores: mean length = 38.5, mean width = 37, mean thickness = 20, Tab. SI 6; flakes: mean length = 38 mm, mean width = 37.3 mm, mean thickness = 13 mm, Tab. SI 13).

The retouched flake/blade tools (Tab SI 4, Fig. SI 12) include scrapers (Fig. 2 n.10), end-scrapers (Fig. 2 n.11), denticulates and notches, that make up most of the tool inventory. Pointed flakes, truncations and becks are present as well.

Whole tools do not generally exceed 81 mm, although two fragments from layer 7 are slightly larger (125 and 94 mm). Metric mean values calculated on 31 whole blanks across layers of 15/1 site are: length = 44.3 mm, width = 33.6 mm, thickness = 12.2 mm (see Tab SI 14 for details on individual layers). Retouched Levallois flakes’ dimensions are overall comparable to those of the unretouched ones (Fig. SI 13).

Common flakes constitute most of the retouched blanks (Tab. SI 15). Some tools are made on predetermined blanks such as blades (ca. 10–33%), laminar flakes (ca. 10%) and Levallois flakes (ca. 10–29%).

Along with the above types, the site also contained one tanged tool made on a thick cortical flake from layer 5 (Fig. 2 n. 12, length = 53 mm, width = 32 mm, thickness = 4 mm), and two backed pieces from layer 4b (Fig. 2 n. 13, length = 36 mm, width = 19 mm, thickness = 13 mm) and layer 4a (Fig. 2 n. 14, length = 37 mm, width = 19 mm, thickness = 7 mm).

At site 16/29 the assemblage is particularly small (124 artifacts, Table SI 4). The Levallois method is testified by end-products and one core for flakes. Dorsal scar pattern on a few Levallois flakes suggests centripetal modes of core reduction (Tab. SI 7). Levallois products do not exceed 79 mm (which also applies to fragments). Mean dimensions, calculated on only three whole specimens, are length = 46 mm, width = 37 mm, thickness = 8.3 mm (Tab. SI 9).

Blades are few, there are no whole specimens, and not much is diagnostic. Nonetheless, the dorsal scar pattern is exclusively unidirectional, and the only recognizable platform is prepared. Maximum length observable among fragments is 29 mm; mean width is 13.5 mm and mean thickness is 3.5 mm.

The Discoid method is identifiable on end-products (Fig. 2 n.16), as well as the Kombewa. Whole specimens (n 2) of the first show mean length = 42.5 mm, width = 50.5 mm, thickness = 13 mm, while the only whole Kombewa flake has length = 30 mm, width = 27 mm; thickness = 9 mm.

These observations further confirm that flake and blade production, whatever the method adopted, was aimed at the production of rather small blanks.

The most common tools (Fig. SI 12) are scrapers, end-scrapers, denticulates (Fig. 2 n. 15) and becks (Fig. 2 n. 20). A fragment of a pointed flake and a tanged tool (Fig. 2 n.19, length = 48 mm, width = 34 mm, thickness = 10 mm) were found in layer 7. Bifacial reduction is proxied by characteristic flakes from layers 3 and 4 (Fig. 2 n. 17, 18). It is also worth signaling the recovery of a point stratified in the same sedimentary sequence ca. 10 m south of site 16/29 (Fig. 2 n. 21, here not included in counts and analyses).

Whole tools do not exceed 92 mm (which also applies to fragments). Metric mean values calculated on 11 whole blanks across layers of 16/29 site are length = 48.1 mm, width = 45 mm, thickness = 15.1 mm (see Tab SI 14 for details on individual layers).

Common flakes constitute most of the retouched blanks (Tab. SI 15). Both Levallois and Kombewa flakes were also used, but with far more limited incidence than common flakes. Retouched Levallois flakes’ dimensions seem comparable to those of the unretouched ones (Fig. SI 13).

## Discussion

**Figure 3:**
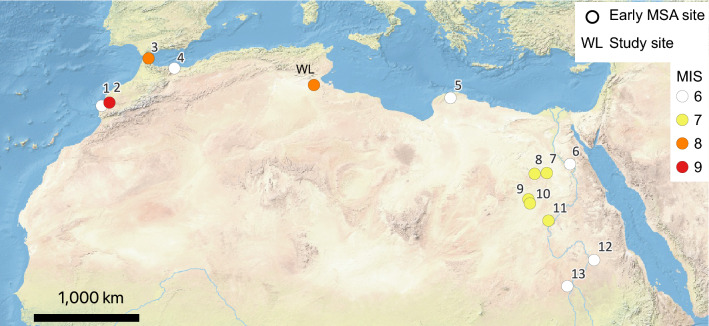
Map of Middle Pleistocene MSA contexts with chronometric estimates in North Africa (WL stands for Wadi Lazalim, the sites here discussed): 1, Bizmoune; 2, Jebel Irhoud; 3, Benzù; 4, Ifri n’Ammar; 5, Haua Fteah; 6, Taramsa 1; 7, Kharga; 8, Dakhla; 9, Bir Tarfawi; 10, Bir Sahara; 11, Sai 8-B-11; 12, EDAR 135; 13, Al Jamrab. Marine Isotopic Stage (MIS) of earliest estimated occupation is also indicated for each site (map created using QGIS 3.20.2 https://qgis.org/it/site/; base map made with Natural Earth).

The investigated contexts at Wadi Lazalim host a stratified archive able to contribute to our knowledge on the emergence of the MSA in the Northern Sahara and in the wider late Middle Pleistocene North African framework. Sites with chronological determinations are extremely few, in particular in the desert regions and in areas suffering socio-political instability. From the Red Sea to the Atlantic Ocean and from the Mediterranean Sea to the Sahel, these documented contexts are rare and only comprise a handful of sites that are far apart and located across thousands of kilometers (Fig. 3).

The archaeological record of the time period here considered is often referred to as Middle Palaeolithic, instead of Middle Stone Age, as a legacy of European research history in North Africa^6,23^. Several scholars have long pointed out that most of the European terminology is not suitable for African contexts^24–26^, also because it introduces misleading distinctions between North Africa and sub-Saharan Africa; as we shall see, this is especially relevant for the case at hand. The early MSA of North Africa comprises several named lithic industries. One of them is the Mousterian, classified as such because of the presence of technological elements like points, notches, sidescrapers or the Levallois technology. The term is in use also today, despite criticism was raised on several occasions (see discussion in e.g.^6,23,27^). The same applies to other terms, i.e. the Sangoan, the Lupemban, the Aterian, whose consistency and usefulness are a continuous matter of debate into which we cannot enter here. We keep using these terms for the sake of clarity and convention.

Site 8-B-11 at Sai Island in northern Sudan preserves one of the rare sequences documenting both Early and Middle Stone Age occupations^28^. In a depression filled with sediments of Middle Pleistocene age in which early Middle Stone Age assemblages are stratified, two lowermost layers are attributed to the Sangoan, while the upper assemblage is qualified as Lupemban^29^. Optically stimulated luminescence (OSL) estimates of < 223 ka, ca. 182 ka and ca. 152 ka frame the time for the early MSA occupation at the site during MIS 7 and MIS 6^28^. Al Jamrab, in the White Nile region in the central Sudan, early MSA assemblages suggesting Sangoan affinities have been identified and provisionally attributed to the late Middle Pleistocene-early Late Pleistocene^30,31^. A large cluster of ESA and MSA stratified contexts was recognized east of the Lower Atbara River in central eastern Sudan^32,33^. OSL dating gave ages for the late Acheulean complexes from before 231 ka, while the early MSA industries recovered in the same area were dated from between 181 and 156 ka^32^. In Egypt, an early MSA occupation in MIS 7 was documented in the oases of the Western Desert dating to ca. 230 ka at Bir Tarfawi and Bir Sahara^34,35^ (see also^36^), and ca. 220 ka at Kharga and Dakhla^37,38^. MIS 6 assemblages dated to around 165 ka, classified as Lupemban, are also known from the Nile Valley at Taramsa-1^39,40^. Early MSA occupation predating the last Interglacial in Libya is documented at Haua Fteah in the Jebel Akhdar, in the northern part of the country, where a little amount of undiagnostic lithic artefacts suggest that humans may have first visited the cave near the end of MIS 6 at around 150 ka^41^. The Maghreb features some of the earliest occurrences of the MSA in Africa, qualifiable as Mousterian, at the Moroccan sites of Jebel Irhoud in MIS 9 at ca. 315 ka^5^ and Benzù in MIS 8 at ca. 250 ka^42^. Middle Stone Age assemblages of later late Middle Pleistocene age (MIS 6) are also found in Morocco at Benzù and at Ifri n’Ammar dating to ca. 170^43^. The late Middle Pleistocene in this region also features early occurrences of the Aterian at Ifri n’Ammar at ca.145^43^ ka and at Bizmoune cave ≥ 142 ka^44^.

While a comparative analysis is far beyond the scope of this paper, some considerations are here drawn based on the evidence unearthed so far at Wadi Lazalim. Here, artefacts lie in secondary position and do not testify to discrete human occupation events, the chronometric determinations thus representing minimum age for the technological setting. A parsimonious scenario for the formation processes of the archaeological record is consistent with low energy surface runoff transport from open air palimpsests located in the rather flat surrounding areas. Artefacts were thus putatively exposed to weathering agents before their eventual burial, after which they underwent further diagenetic processes affecting them differentially according to raw materials’ type.

As globally suggested by the technology and size of cores, blanks and tool types, the archaeological record comfortably fits a widely accepted concept of an MSA framework. The lithic assemblages are mostly flake and blade-based. The recurrent Levallois is the most observed reduction method, but Levallois point reduction was also observed. Laminar production was also widely identified. Discoid and Kombewa are attested as well. All of these reduction methods were invariably aimed at the production of rather small predetermined blanks, which do not seem be influenced by the available raw material, whose quality and size would have allowed a wide range of production sequences. The technological choices were thus little (or not) constrained by the local natural lithic resources. The assemblages mostly include rather common tool types such as scrapers, end-scrapers, denticulates, and pointed flakes, but also comprise elements that are far more rarely found in late Middle Pleistocene North African MSA assemblages, such as bifacial tools, backed tools and tanged tools.

No “genuine” Aterian tanged tools has been found so far within the excavated materials. Conversely, quite typical Aterian points have been found on top of the deflated stone pavements above the investigated sequences. As the series of luminescence dating first firmly shows, this observation further supports the attribution of the unearthed materials to early MSA phases predating the Aterian. Based on a few well dated contexts, the Aterian is commonly accepted to date from the MIS 5/6 boundary up until MIS 2^45^.

Although intermixing of materials from different occupation events no doubt occurred, the three depositional sequences provided coarse but coherently ordered proxies for late Middle Pleistocene human activity and related technological signature in a way not so dissimilar from other – exceptionally rare—late Middle Pleistocene MSA stratified contexts in Africa. An example is Kalambo Falls (Zambia), where fluvial activity was recognized as the main driver of the deposition, yet the late Acheulean and early MSA Sangoan and Lupemban archaeological record are thought to still retain a solid chronostratigraphic coherence^46,47^.

Similarly, the three investigated sequences at Wadi Lazalim are distributed in a coherent chrono-stratigraphic succession spanning the late Middle Pleistocene from at least MIS 8. Their archaeological content also show a seemingly consistent chrono-cultural diachronic succession. As discussed in what follows, this is testified for instance by the large bifacial component, which is restricted to the earliest among the three sites (16/15), whereas other technologies including backed pieces, points and tanged tools show up at later 15/1 and at 16/29 sequences.

Layer J at site 16/15, which directly rests on the bedrock (K), is the earliest evidence in our data-set. It includes Levallois flakes (Fig. SI 14) and blades, scrapers and denticulates; a large roughout, likely for a bifacial tool, equally comes from this layer, for which an attribution to the Acheulean cannot be excluded. Stronger elements in favor of this possibility are nevertheless currently lacking. The overlying layer I has a rich assemblage with Levallois (Fig. SI 14), Discoid and Kombewa flakes, blades, abundant scrapers, denticulates and end-scrapers. Based on the date from the base of overlying layer H (Fig. SI 15), layer I is conservatively older than 250 ka. Other than a likely comparable chronology, the archaeological record from layer I seemingly shares most of the reduction methods and tool kits with the earliest North African MSA occurrences at Jebel Irhoud and Benzù, which are “Mousterian” assemblages characterized by Levallois technology, side scrapers, end scrapers, denticulates, retouched points^7^. Any evidence for bifacial shaping is anyhow lacking at these Maghrebian early contexts, which would prevent further matching with the above layer H, whose base is likely older than MIS 8 and returned a flake industry including Levallois flakes and denticulates as well as bifacial knives and a core-axe (as defined by^22^). The inclusion of such large cutting/heavy-duty tools within an overall “Mode 3” technological milieu naturally might suggest affinities with the Sangoan of Northeast Africa, where core-axe manufacture anyhow occur in assemblages where discoid and blade reduction are also practiced^29^.

The remaining part of the sequence at site 16/15 remains for the moment less informative: layer G, whose dating result overlaps with the date of the underlying layer H (because of large standard errors), returned Levallois flakes, blades and a few tools; layers above G (BC, DEF, A) yielded assemblages of an MSA attribution with Levallois flakes, blades and retouched tools whose chronological position is currently not known.

At site 15/1 the basal layer 11 (undated) is archaeologically sterile, as well as top layers 1–3. According to the dating results, these top layers could date from the end of MIS 7 or the onset of MIS 6. Archaeological materials are found in the group of layers comprised between 4a and 9/10, for which the chronometric determinations (4a = 252 ± 23 ka; 4b = 232 ± 22 ka; 5 = 234 ± 22 ka; 9/10 = 207 ± 21 ka) are attributable to MIS 7–8. The density of lithic artefacts at this site is particularly low. The lithic assemblages are mostly flake-based (Levallois, Discoid and Kombewa). Laminar production was also widely observed. Along with scrapers, end-scrapers, denticulates, retouched points, the assemblages revealed Levallois point reduction, backed pieces and a crudely tanged artefact standing among the main distinguishing features, for they are very infrequently found in late Middle Pleistocene North African contexts^6^. Backed pieces from site 15/1 could find some parallels at a similar chronological framework with the Sangoan and Lupemban *tranchets* at Sai 8-B-11 ^29,48^, while the earliest North African tangs from Ifri N’Ammar^43^ and Bizmoune Cave^44^ would postdate the one found at Wadi Lazalim 15/1.

The chronological determinations at site 16/29 layer 3 (149 ± 15 ka), layer 5 (131 ± 13 ka) and layer 6 (160 ± 14 ka) frame its archaeological content mostly in MIS 6. Layers 1 and 2 did not return artefacts, as well as layer 8. The assemblages consist of flake-based industries characterized by Levallois reduction. Discoid and Kombewa are represented as well. Common tool types are scrapers, end-scrapers, denticulates and becks. The shaping of bifacial tools is here proxied by characteristic small shaping flakes. The finding of a bifacial point, most likely a roughout, included in sediments a few meters apart, supports the likelihood that such tools were equally part of the technological inventory. A crude tang in layer 7 and the dating of overlying layer 6 to 160 ± 14 ka would also in this case push back in time the occurrence of tanging recognized so far in North Africa^43,44^.

## Conclusion

Wadi Lazalim revealed early MSA hominin occupation of the northern Sahara during a time close to that of the emergence of the MSA itself, very cautiously allowing the inclusion of this area into narratives around the onset of the MSA in North Africa. The earliest technological pattern identified at site 16/15, putatively MIS 8 or earlier, finds the closest connections, also on chronological grounds, with the earliest MSA record of the fairly close NW Maghreb, which can be described as basically made of a “Mousterian” substratum, virtually shared by most of North African early MSA assemblages^6^. It is yet unclear and it is not currently possible to determine with some confidence whether these very early occurrences in NW Africa, which are also among the earliest in the African continent, represent the outcome of multiple and interrelated regional developments (from e.g. the local Acheulean) or of diffusion processes from areas of endemism where changes in behavior and technology possibly occurred at an earlier age^5^. The later findings from Wadi Lazalim, especially those of a likely MIS7 age from site 16/15-layer H and from site 15/1-layers 4a-5, might suggest slightly more circumstantial connections with the wider early MSA African framework. They comprise in fact technological elements that, in North Africa, were either unknown during this timeframe (the tanged tools), or only identified (core-axes and backed tools) in Sangoan and Lupemban technocomplexes in the Nile Valley and adjacent deserts^2,29^. Not unanimously agreed, the identification of such industries in North Africa long suggested diffusion processes of early MSA components from central Africa^28^, confirming the importance of this latter region for human origins^49^. In these regards, it has to be said that Sangoan and Lupemban are persistently regarded as a regional adaptation to central Africa tropical forests; yet the coarse paleoecological and chronological evidence does not warrant such direct correlation^50^. It suggests instead they may cautiously be related to the exploitation of a wide range of environments, including grasslands, woodlands and forests, for which especially the Lupemban composite-tool technology, with its foliates, backed tools and tanged tools^51^, provided an effective and flexible adaptive means^52^. Backed tools, for example, are considered a technological means to counter the risk of being under-equipped during explorative displacements across unfamiliar territories^53^ or for resources acquisition in rapidly changing environments imposing tuning of technology, subsistence-settlement system and economic strategy^54,55^.

On a chronological and technological ground, data from Wadi Lazalim might thus suggest south to north trans-Saharan diffusion of early MSA elements in the framework of increasingly variable ecological conditions of the late Middle Pleistocene^56,57^, which could have been triggers of broader acquisition spectra, increased interactions between populations, improved social networks^58^ and circulation of technological innovations. Pulses of ecological continuity with the Mediterranean could have also provided cyclically viable windows of opportunity for diffusion processes^9^.

Using similarities in stone tool technologies for inferring diffusion of technological elements can be misleading, because it should always be taken into account the possibility of independent innovation^59^. However, the coarse resolution of the chronological and environmental framework of the earliest phases of the MSA does not allow to neither safely accept nor reject the alternative hypotheses (diffusion VS convergence) that could explain such similarities. In any cases, especially if complex strategies like composite tool technology are considered, and the sub-continental scale discussed here, diffusion seem quite more parsimonious than systematic convergence phenomena. Moreover, although from Late Pleistocene MSA frameworks much later than those herein discussed, the recently demonstrated long range transbiome diffusion through networking of ostrich eggshell beads in Eastern and Southern Africa^60,61^ warns that scales of magnitude in the order of thousands of kilometers should not prevent to at least hypothesize connections between areas far apart considerable distance.

To conclude, this is the very first time that in North Africa we see something comparable to sub-Saharan early MSA outside the Nile Valley in a comparable time frame. Although it is not here suggested a Sangoan/Lupemban occurrence in the northernmost edge of the Sahara, it is suggested that at least part of the early manifestations of the MSA in North Africa could be considered as an outcome of the dispersion of technological elements from sub-Saharan Africa that were likely filtered, adjusted, modified “while in the Sahara” to an extent we don’t currently perceive if not at a very coarse level^2,62,63^. At the very least, the data from Wadi Lazalim adds to the complexity of the general picture by providing support to long held assumptions that variability of MSA assemblages in North Africa firmly depends also on sub-Saharan influx. Moreover, since human fossils of late Middle Pleistocene age in North Africa are almost restricted to the Jebel Irhoud ones, the most part being of Late Pleistocene age^64^, understanding human biogeography in this area remains speculative and strongly relies upon supposed convergences with the material culture. Although considering artefacts as proxies for human types is deceptive, the recognized co-evolution of the MSA and our species^4,5^ encourages the inclusion of areas and sites without human fossils into the current *H. sapiens* biogeographic narrative, for which the reported data from Wadi Lazalim represent a strong contribution.

## Methods

### The excavations

The excavation of test trenches at Sites 15/1, 16/29 and 16/15 (Site 15/1: 2 m wide and 5.2 m deep, bedrock reached; Site 16/29: 1 m wide and 2.4 m deep, bedrock not reached; Site 16/15: 2 m wide and 2.1 m deep, bedrock reached) (Fig. 1) were primarily aimed at obtaining geochronological, sedimentological and archaeological data from a sequence as long as possible. Excavation was performed according to stratigraphic units identified and differentiated based on field sedimentological observations, which included texture, color, type of matrix, presence and size of stone clasts, bioturbation. Thicker layers were subdivided into artificial spit layers. Most of the deposits are very hardened, cemented, and badly processable with small tools in a reasonable time. The excavation was thus performed mostly by manual tools like hand picks and chisels. Sediments were entirely dry sieved (mesh 4 mm) to collect archaeological debris. The positioning of artifacts was surveyed using an Electronic Total Station (Leica TCR307).

### Sediment sampling for IRSL dating

Sediments were sampled for luminescence dating from the profiles exposed during the 2016 field mission, using either lightproof metal pipes inserted into the cleaned stratigraphic sections or by collecting whole blocks, depending on the type of sediment being sampled. To prevent light exposure, the metal pipes used for sampling were already closed at one end by a welded metal plate, while the other end was closed with a plastic plug and then wrapped with adhesive tape immediately after removing the pipe. Blocks were extracted by carving cubes of indurated sediment with a side length of at least 10 cm and then wrapped into aluminum foil and adhesive tape.

Eleven sediments collected in the 16/29 (n = 3), 16/15 (n = 2) and 15/1 (n = 6) sites have been dated using infrared stimulated luminescence (pIRIR_290_) at the IRAMAT-CRP2A laboratory (Bordeaux Montaigne University, France). Sediment preparation and measurements were conducted in the laboratory under subdued red or orange light. The sediment were extracted from the blocks and the pipes, discarding the more external parts that were possibly exposed to light during sampling.

Both samples IRSL 17 and IRSL 16 from Site 16/15 (Fig. SI 3) were collected by hammering metal pipes into the sandy-silty sediments and correspond to the base of layer H and the top of layer G, respectively.

At Site 15/1 (Fig. SI 3), only layer 3 allowed collecting a sediment sample by using a metal pipe (sample IRSL 1), because other layers were very indurated and/or contained stone clasts, we removed whole blocks. Sample IRSL 7 was collected at the top of layer 1, which is the gypsum crust. Sample IRSL 6 was collected at the top of layer 4a, immediately below the base of layer 3. Samples IRSL 4 and 5 were collected by removing a single block including the base of layer 4b and the top of layer 5. The block was then split in two portions along the interface between the two layers. Sample IRSL 3 was collected carving a block from the middle portion of layer 9/10.

The deposit of Site 16/29, although very indurated, allowed the collection of sediment samples using metal pipes (Fig. SI 3). Samples IRSL 14, IRSL 8 and IRSL 15 were collected near the base of layers 3, 5 and 6, respectively.

### IRSL dating protocol

The sediment samples collected were sieved and the most dominant granulometric fraction (100–120 µm) was selected. This fraction was treated with HCl (10%) to dissolve carbonates and H_2_O_2_ (30%) to degrade organic matter, then divided into two sub-fractions. The first one was treated with H_2_SiF_6_ to dissolve feldspars and isolate quartz grains. Preliminary tests on multigrain quartz aliquots, using a Freiberg Smart reader equipped with green emitting diodes^65^, indicated a saturation of the OSL signal (Fig. SI 4). Therefore, density separation (d = 2.565) was completed on the second sub-fraction to extract potassium rich feldspar (K-feldspar) grains. The IRSL signal from feldspar grains was then used for equivalent dose determination since this signal allows extending the dating limit up to 500 ka^66^. IRSL analyses using the pIRIR_290_ protocol^67^ were performed on feldspar since the signal measured at 290 °C does not experience from anomalous fading. Single grain analyses were undertaken on on a TL/IRSL DA20 Risø reader (Table S1).

An infrared laser was used for the stimulation and a combination of optical filters for signal detection (Schott BG39 + Corning 7–59). For all analyses, equivalent doses (D_e_) were determined in applying a SAR protocol^68^.

In the absence of dosimetric measurements in situ, a fraction of sediment was analyzed to determine the environmental dose rate. In the laboratory, it was dried for a week (40 °C) and homogenized before analyzing a fraction of ~ 100 g with a gamma-ray spectrometer using a high-resolution, broad-energy Ge detector. By comparison with a standard, the U, Th and K contents of each sample were then determined (Table SI 2). The external dose rates (α, β and γ) deriving from these contents were calculated using the conversion factors of Guérin et al. ^69^, and the cosmic dose contribution calculated considering the sediment cover^70^. Regarding the internal dose rate, the potassium content was measured in feldspar grains using SEM–EDS. A mean value and standard deviation was calculated specifically for each sample (Table SI 2).

Single grain IRSL data were processed using Analyst v.4.52^71^. The signal was integrated using the first 0.05 s and the background was subtracted from the last 0.25 s. The following criteria were applied for D_e_ selection: a recycling ratio limit of 10%; a maximum recuperation of 5% of the natural signal; a maximum test dose error of 10%; a test dose signal more than 3 sigma above background. D_e_ higher than two times D_0_ were discarded^72^.

The residual dose was measured after bleaching nine aliquots of BDX 19037 (15/1), 19042 (16/29) and 19044 (16/15) in a solar simulator (Hölne SOL500) for 30 min, 25 h and 95 h. Mean values and standard deviations were calculated on aliquots bleached for 95 h for BDX 19037 (9.18 ± 4.78 Gy), 19042 (7.53 ± 3.37 Gy) and 19044 (8.97 ± 0.84 Gy) and subtracted to the D_e_ obtained on samples from sites 15/1, 16/29 and 16/15 respectively.

A large scatter of the individual pIRIR_290_ D_e_ values is observed (Fig. SI 5) but no particular shape of the distribution (which, for instance, might indicate distinct grain populations bleached at different levels during transportation and deposition) can be identified. The Central Age Model (CAM)^73^ was then applied to calculate the mean D_e_ value for each sample. These mean values increase systematically with depth, except in Site 16/15 where the two samples lead to indistinguishable values despite an elevation difference of about 60 cm (Table SI 3).

Ages were calculated with a 1 σ error range (Table SI 3) considering beta absorption factors of 0.10 (U), 0.14 (Th) and 0.04 (K)^74^; alpha attenuation factors of 0.15 (U) and 0.18 (Th)^75^; an α-value of 0.08 ± 0.02^76^, and a water content of 3% in the sediment.

The obtained IRSL ages follow the stratigraphic order, considering the error range. Such results do not allow differentiating occupation phases; they might reflect the poor representativity of the sediment samples used for determining the U, Th, K contents, and then an inaccurate assessment of the environmental dose rate. Nevertheless, the ages span the second half of the Middle Pleistocene and potentially the beginning of the Upper Pleistocene.

## Data availability

All data is available in the manuscript or the Supplementary Information.

## Supplementary Information

Supplementary Information.
