# Supplementary material for: A late Middle Pleistocene Middle Stone Age sequence identified at Wadi Lazalim in southern Tunisia
Source: Sci Rep. 2022 Mar 18;12:3996. doi: 10.1038/s41598-022-07816-x (PMC8933421; doi:10.1038/s41598-022-07816-x)
Supplement: Supplementary file 1 — Supplementary Information. [file 41598_2022_7816_MOESM1_ESM.pdf]

**Supplementary Information**

A late Middle Pleistocene Middle Stone Age sequence identified at Wadi Lazalim in southern Tunisia

Emanuele Cancellieri[1*], Hedi Bel Hadj Brahim[2], Jaafar Ben Nasr[3], Tarek Ben Fraj[4,5], Ridha Boussoffara[6], Martina Di Matteo[1], Norbert Mercier[7], Marwa Marnaoui[3], Andrea Monaco[1], Maïlys Richard[7,8], Guido S. Mariani[9], Olivier Scancarello[1], Andrea Zerboni[10], Savino di Lernia [1,11]

[1] Dipartimento di Scienze dell'Antichità, Sapienza University of Rome, Italy

[2] Artisanat du Sahara, Douz, Tunisia

[3] Faculté des Lettres et des Sciences Humaines, Université de Kairouan, Tunisia

[4] Faculté des Lettres et des Sciences Humaines, Université de Sousse, Tunisia

[5] Laboratoire de Cartographie Géomorphologique des Milieux, des Environnements et des Dynamiques (CGMED), Université de Tunis, Tunisia

[6] Institut National du Patrimoine, Tunis, Tunisia

[7] Archéosciences-Bordeaux, UMR 6034 CNRS-Université Bordeaux Montaigne, Pessac, France

[8] Centro Nacional de Investigación sobre la Evolución Humana (CENIEH), Burgos, Spain

[9] Dipartimento di Scienze Chimiche e Geologiche, Università degli Studi di Cagliari, Italy

[10] Dipartimento di Scienze della Terra "A. Desio", Università degli Studi di Milano, Italy

[11] School of Geography, Archaeology and Environmental Studies, University of the Witwatersrand, Johannesburg, South Africa

* correspondence: ema.cancellieri@gmail.com

*The excavations*

The research area was first identified during a brief field mission conducted in 2015 along the Wadi Lazalim, ca. 40 km east of the town of Douz. Field research in the following two seasons (2016-2017) first consisted in intensive surveys along the main course of the wadi and along its tributaries to check the sedimentary successions. The survey was followed by the excavation of three test trenches at localities where artifacts were visible along fluvial cuts to evaluate the archaeological potential of the area: at Site15/1 along the left bank, at Site 16/29 along the right bank a few hundred meters upstream of Site 15/1, and at Site 16/15, along the right bank of a small tributary (Fig. SI 1).

The excavations were primarily aimed at obtaining geochronological, sedimentological and archaeological data from diverse localities. We have dug trenches through the depth of the selected sites (Site 15/1: 2 m wide and 5.2 m deep, bedrock reached; Site 16/29: 1 m wide and 2.4 m deep, bedrock not reached; Site 16/15: 2 m wide and 2.1 m deep, bedrock reached). Most of the deposits are very hardened, cemented, and badly processable with small tools. The excavation was thus performed mostly by manual tools, like hand picks and chisels. Sediments were entirely dry sieved (mesh 4 mm) to collect most part of the archaeological debris. The positioning of artifacts was recorded by means of an Electronic Total Station (Leica TCR307).

Excavation was performed according to stratigraphic units identified and differentiated because of field sedimentological observations, which included texture, color, type of matrix, presence and size of stone clasts, bioturbation. Thicker layers were subdivided into artificial spit layers (15 cm).

At Site 15/1, a step trench one-meter wide was first dug in 2016 along the left bank of the wadi in correspondence with the area where lithic artefacts were visible in the profile. The surface of the excavation was kept to a maximum of 1 $m^2$, from the top of the first small terrace corresponding to the base of layer 3. The resumption of the excavations at Site 15/1 in 2017 was primarily aimed at reaching the base of the sequence, not reached during the previous field season. We first excavated an area of about 5 x 5 m with a small bulldozer directly above the trench, on the plateau, to quickly

remove the thick gypsum crust (layer 1) and the underlying sterile silty deposits (layer 3). A maximum area of 3 x 4 meters was delimited and subdivided into squares A-D/1-3. The excavation was then performed manually from top of layer 4a.

At Site 16/29, a step trench one-meter wide was first dug in 2016 along the right bank of the wadi in correspondence with the area where lithic artifacts were visible along the section. The search investigated the entire sequence' for about 2 m. In 2017 we excavated portions of the deposit left at the end of the 2016 mission (the steps), from layer 4 to the base of the sequence (layer 8). The maximum excavated area was 2 $m^2$, subdivided into squares A 1-2.

At Site 16/15, a half a meter-wide trench was first excavated in 2016 along the left bank of a small tributary of wadi Lazalim for a total depth of ca. 1.5 m in an area of very high abundance of archaeological materials visible through the sequence. The trench was kept to a very small size because of the little time available that year. The trench was then widened in 2017 to 2x3 m, for a total excavation depth of ca. 2 meters. The excavation surface was subdivided into six squares (A-B/1-3).

*Description of the sedimentary sequences*

Site 16/15

The 2.1 m deep trench excavated along the first erosion glacis at Site 16/15, along the right bank of a tributary of Wadi Lazalim, ca. 700 m eastward the main course of the wadi (Fig. SI 1), exposed 11 sedimentary units separated by erosional surfaces (Main Text Fig. 1). The surface layer A is a bioturbated unconsolidated coarse deposit. Layers B-C are friable to moderately consolidated deposits with few angular gravels in a sandy-silty matrix. Layers D-F are moderately consolidated sandy-silty deposits with rare clasts. Layer G is a consolidated silty sandy deposit with subangular blocks, pebbles and gravels. The sediment sample from this layer returned an age of 357 ± 52 ka (MIS 11-9), which falls within the same range (considering the errors at 1σ) than the age of 285 ± 32 ka (MIS 8-9) obtained for the base of the underlying layer H, a consolidated deposit of sand and silt containing rare pebbles and gravels. The lowermost layers J and I are well consolidated coarse deposits of sub-angular blocks and pebbles within a sandy matrix. They rest directly on the bedrock composed of white limestone dislocated in blocks. Overall, blocks with a size greater than 20 cm represent ~2% of the sediments. Small and medium blocks (10 to 20 cm) represent ~3%, while gravels and pebbles (less than 10 cm) represent ~10%.

Site 15/1

The trench excavated in the contact between the second erosion glacis and the substitution glacis, developed following the partial destruction of the second glacis at Site 15/1, along the left bank of Wadi Lazalim (Fig. SI 1), exposed 9 sedimentary units on a total height of 5.2 m separated by erosional surfaces (Main Fig. 1D). The top layer 1, a thick gypsum crust, returned an age of 217 ± 39 ka (MIS 8-6). Layer 3, archaeologically sterile, is a hardened deposit of centimetric slightly wavy layers of pebbles within a matrix of sand, silt and clay, dated at 177 ± 19 ka (MIS 6-7). Artifactual evidence starts with layer 4a, a moderately hardened coarse deposit, formed by blocks

and pebbles in a matrix of sand, silt and clay dated at 252 ± 23 ka (MIS 8-7), very similar to underlying layer 4b, dated at 232 ± 22 ka (MIS 8-7), from which is separated by a discontinuity. Layer 5, dated at 234 ± 22 ka (MIS 8-7) is a moderately hardened coarse deposit with subangular clasts in a matrix of silt, sand and clay. Layer 6 is a thin friable deposit of small subangular pebbles and gravels in a sandy matrix. Layer 7 is a moderately hardened coarse deposit of mostly subangular pebbles and gravels within a matrix of sand and clay. Layer 8 is a discontinuous thin friable lens of silt and clay very rich in gypsum. Layer 9/10 is the lowermost artifact-bearing layer. It is a coarse moderately hardened deposit made of pebbles, gravels and small subangular blocks in a matrix of sand, silt and clay. The age for this layer is 207 ± 21 ka (MIS 6-7). The lowermost unit is archaeologically sterile layer 11, made of clays with silts in a polygonal structure. Blocks of 10-20 cm in diameter represent ~10 % the sediment matrix, while gravel stones less than 10 cm make up ~25 %.

Site 16/29

The trench excavated in the second erosion glacis at Site 16/29, along the right bank ca. 300 meters upstream of 15/1 (Fig. SI 1), exposed 8 sedimentary units on a height of 2.4 m (Main Fig. 1D). Layer 1 is the gypsum crust. Layer 2 is a thin horizon of white loose gypsum crystals. Layer 3 is a moderately consolidated deposit containing small subangular gravels and pebbles in a matrix of sand and silt; it was dated to 149 ± 15 ka (MIS 6). Layer 4 is a moderately consolidated heterometric deposit of subangular blocks, pebbles and gravels arranged in bulk contained in a matrix of sand and clay. Layer 5 is a very indurated sandy clayey deposit rich in crystallized gypsum containing small pebbles and is dated to 131 ± 13 ka (MIS 6-5). Layers 6 and 7 are moderately consolidated deposits of sand and clay containing sub-rounded blocks and pebbles. An age of 160 ± 14 ka (MIS 6) is assigned to layer 6. Layer 8 is a very consolidated deposit made of sand, silt and clay with small blocks. Blocks of 10-20 cm in diameter represent ~6 % of the sediment matrix, while gravel stones less than 10 cm make up ~15 %.

*Grain-size analysis*

Grain size compositions of the three studied sections show considerable similarities (Fig. SI 6). Sand represents the most common fraction in the sediment of Site 16/15 with values between 40 and 70%. It is also prevalent in the lower portion of Site 16/29, where it increases with depth from 40 to 60%. An in-depth look at size classes allows attributing most of these values to the fine portions of sand: medium to coarse size classes are sporadic in the deposit, and often completely absent. Silt is instead the main component of the upper portion of Site 16/29: here it decreases in frequency with depth, passing from 60 to 30%. It is also the main component of Site 15/1, where it varies with depth between 40 and 75%. Clay is always scarce, never higher than 10% in any of the three sections. Trends in grain size along the sections are present, but always gradual and without obvious abrupt changes. Site 16/29 is the most monotonous and shows a simple trend of silt decrease and sand increase with depth. Site 16/15 on average has no significant trends: although variations between close samples can be high (even greater than 10%) the proportions between the various components remain mainly unchanged considering the whole section. This is partially different if we consider the sand components, with the abrupt appearance of coarse sand from 100 cm downwards. Nevertheless, this fraction is always smaller than 10% of the total volume, and the rest of the components remain mostly constant. Site15/1, the deepest section, shows several changes in grain size compositions, alternating sand and silt as the dominant class. However, these variations are always gradual, without abrupt shifts. Coarse sand fractions appear between 320 and 500 cm, but even this trend is gradual and quite small. Most of the sequences shows the juxtaposition of coarse sediments (gravel and lithics) interspersed in a matrix constituted by fine sediments with different percentages of sand, silt, and clay. Such evidence suggest that sedimentation occurred at different stages controlled by slope processes and denudation of surrounding pediments. We thus suggest that colluvial process is the major player in the formation

of stratigraphic sequences.  Colluviation (occasionally alternated with alluvial processes) occurred in the Middle and Upper Pleistocene, alternated to erosional phases and occasional deactivation of surface processes. Superimposed to such processes weak to moderate soil-forming processes took place; pedogenesis become stronger in the uppermost part of sequences, where increased evapotranspiration lead to the huge accumulation of gypsum.

**Table SI 1:** pIRIR$_{290}$ SAR measurement protocol used in this study.

| Step | Treatment |
|---|---|
| 1 | Given dose |
| 2 | Preheat (320°C for 60 s) |
| 3 | IR stimulation for 200 s at 50°C |
| 4 (Lx) | SG IRSL stimulation for 2 s at 290°C |
| 5 | Given test dose |
| 6 | Preheat (320°C for 60 s) |
| 7 | IR stimulation for 200 s at 50°C |
| 8 (Tx) | SG IRSL stimulation for 2 s at 290°C |
| 9 | IRSL stimulation for 60 s at 325°C |
| 10 | Return to 1 |

**Table SI 2:** Radioelement contents of sediment samples (used to derive external dose rates) and in K-feldspar grains (used to derive internal dose rates).

[1] Radioelement contents determined using gamma-ray spectrometry

[2] K content measured in feldspar grains using SEM-EDS.

| Lab # | Sample # | Site | Layer | External radioelement content[1] | | | Internal K (%)[2] |
|---|---|---|---|---|---|---|---|
| | | | | $^{238}U$ (ppm) | $^{232}Th$ (ppm) | $^{40}K$ (%) | |
| 19036 | IRSL 7 | 15/1 | 1 | 0.333 ± 0.008 | 0.419 ± 0.015 | 0.083 ± 0.004 | 12.6 ± 1.2 |
| 19035 | IRSL 1 | 15/1 | 3 | 1.647 ± 0.018 | 3.038 ± 1.037 | 0.592 ± 1.004 | 11.3 ± 1.5 |
| 19039 | IRSL 6 | 15/1 | 4a | 0.988 ± 0.013 | 1.721 ± 0.026 | 0.353 ± 0.004 | 11.6 ± 1.6 |
| 19037 | IRSL 4 | 15/1 | 4b | 1.698 ± 0.016 | 1.557 ± 1.022 | 0.300 ± 1.004 | 12.7 ± 1.5 |
| 19038 | IRSL 5 | 15/1 | 5 | 1.288 ± 0.015 | 1.972 ± 1.029 | 0.451 ± 1.004 | 12.2 ± 1.8 |
| 19043 | IRSL 3 | 15/1 | 9/10 | 1.417 ± 0.016 | 3.172 ± 1.037 | 0.579 ± 1.004 | 11.1 ± 1.5 |
| 19041 | IRSL 14 | 16/29 | 3 | 0.607 ± 0.010 | 0.825 ± 0.018 | 0.171 ± 0.004 | 12.5 ± 1.4 |
| 19040 | IRSL 8 | 16/29 | 5 | 1.389 ± 0.015 | 2.893 ± 1.035 | 0.521 ± 1.004 | 8.9 ± 2.1 |
| 19042 | IRSL 15 | 16/29 | 6 | 1.613 ± 0.017 | 2.959 ± 1.036 | 0.542 ± 1.004 | 11.3 ± 1.6 |
| 19044 | IRSL 16 | 16/15 | G | 0.331 ± 0.008 | 0.651 ± 0.017 | 0.128 ± 0.004 | 9.5 ± 1.9 |
| 19045 | IRSL 17 | 16/15 | H | 0.595 ± 0.011 | 1.030 ± 0.021 | 0.195 ± 0.004 | 10.7 ± 2.1 |

**Table SI 3:** pIRIR$_{290}$ ages obtained on this study.

| Lab. # | Sample # | Site | Layer | Depth (cm) | De (Gy) | Dose rate ($\mu$Gy/a) | | | | | | Age (ka) |
|---|---|---|---|---|---|---|---|---|---|---|---|---|
| | | | | | | Alpha | Beta | Gamma | Cosmic | Int. Beta | Total | |
| 19036 | IRSL 7 | 15/1 | 1 | 30 | 176 ± 29 | 12 | 113 | 75 | 219 | 392 | 811 ± 58 | **217 ± 39** |
| 19035 | IRSL 1 | 15/1 | 3 | 130 | 313 ± 25 | 65 | 716 | 461 | 176 | 352 | 1770 ± 129 | **177 ± 19** |
| 19039 | IRSL 6 | 15/1 | 4a | 160 | 319 ± 16 | 38 | 426 | 271 | 169 | 361 | 1265 ± 96 | **252 ± 23** |
| 19037 | IRSL 4 | 15/1 | 4b | 230 | 326 ± 20 | 54 | 473 | 328 | 154 | 395 | 1404 ± 103 | **232 ± 22** |
| 19038 | IRSL 5 | 15/1 | 5 | 235 | 342 ± 20 | 48 | 542 | 339 | 153 | 380 | 1461 ± 108 | **234 ± 22** |
| 19043 | IRSL 3 | 15/1 | 9/10 | 330 | 344 ± 24 | 61 | 680 | 439 | 135 | 346 | 1661 ± 122 | **207 ± 21** |
| 19041 | IRSL 14 | 16/29 | 3 | 60 | 145 ± 8 | 22 | 222 | 145 | 194 | 389 | 972 ± 81 | **149 ± 15** |
| 19040 | IRSL 8 | 16/29 | 5 | 145 | 202 ± 11 | 58 | 628 | 409 | 172 | 277 | 1544 ± 127 | **131 ± 13** |
| 19042 | IRSL 15 | 16/29 | 6 | 190 | 271 ± 13 | 64 | 673 | 442 | 162 | 352 | 1692 ± 122 | **160 ± 14** |
| 19044 | IRSL 16 | 16/15 | G | 65 | 268 ± 22 | 13 | 95 | 97 | 193 | 296 | 750 ± 89 | **357 ± 52** |
| 19045 | IRSL 17 | 16/15 | H | 125 | 267 ± 17 | 23 | 243 | 159 | 177 | 333 | 935 ± 86 | **285 ± 32** |

**Table SI 4:** Count of lithic artefacts from sites 16/15, 15/1, 16/29 classified according to site, stratigraphic provenance, technology and typology.

| Cathegory | N of artefacts per layer or groups of layers | | | | | | | | | | | | | | | | | | | | | | | | | | |
|---|---|---|---|---|---|---|---|---|---|---|---|---|---|---|---|---|---|---|---|---|---|---|---|---|---|---|---|
| | Site 16_15 | | | | | | | | | Site 15_1 | | | | | | | | | Site 16_29 | | | | | | | Total | % |
| | A | BC | DEF | G | H | I | J | Total | % | 4a | 4b | 5 | 6 | 7 | 8 | 9-10 | Total | % | 3 | 4 | 5 | 6 | 7 | Total | % | | |
| Undeterminable | | | | | | | | | | | | | | | | | | | | | | | | | | | |
| Undeterminable | | | | | | | | | | 1 | 3 | 5 | | 3 | | | 12 | 4.1 | 1 | 1 | 2 | 1 | | 5 | 40 | 17 | 15 |
| Flake | | | | | | | | | | | | | | | | | | | | | | | | | | | |
| Flake | 87 | 63 | 25 | 32 | 62 | 240 | 52 | 561 | 77.9 | 19 | 21 | 52 | 10 | 29 | 8 | 33 | 172 | 59.1 | 5 | 30 | 3 | 23 | 13 | 74 | 59.7 | 807 | 71.1 |
| Biface shaping flake | | | | | | | | | | | | | | | | | | | 1 | 1 | | | | 2 | 16 | 2 | 02 |
| Management flake (core trimming element) | 3 | | 1 | 1 | | 1 | | 6 | 08 | | 1 | 1 | | 1 | | 5 | 8 | 2.7 | | | | 1 | | 1 | 08 | 15 | 13 |
| Flake total | 90 | 63 | 26 | 33 | 62 | 241 | 52 | 567 | 78.8 | 19 | 22 | 53 | 10 | 30 | 8 | 38 | 180 | 61.9 | 6 | 31 | 3 | 24 | 13 | 77 | 62.1 | 824 | 72.6 |
| Predetermined blank | | | | | | | | | | | | | | | | | | | | | | | | | | | |
| Blade | 5 | | | 4 | 1 | 5 | 2 | 17 | 2.4 | 1 | 2 | 4 | | 2 | | | 9 | 3.1 | | 3 | | | 1 | 4 | 32 | 30 | 2.6 |
| Burin spall | | | | | | 1 | | 1 | 0.1 | | 1 | | | | | 1 | 2 | 0.7 | | | | | | | | 3 | 03 |
| Levallois flake | 5 | 4 | 1 | 3 | 7 | 16 | 7 | 43 | 6.0 | 1 | | 1 | | 1 | | 2 | 5 | 1.7 | | 2 | | 1 | 1 | 4 | 32 | 52 | 4.6 |
| Levallois point | | | | | | | | | | | 1 | | | | | | 1 | 03 | | | | | | | | 1 | 0.1 |
| Discoid flake | | | | | | 1 | | 1 | 0.1 | | 2 | | | 2 | | | 4 | 1.4 | | 1 | | 1 | | 2 | 1.6 | 7 | 06 |
| Kombewa flake | | | | | | 2 | | 2 | 03 | | 1 | | | | | 1 | 2 | 0.7 | | | | 2 | | 2 | 1.6 | 6 | 05 |
| Laminar flake | | | | | | | | | | | 2 | | | | | | 2 | 0.7 | | | | | | | | 2 | 02 |
| Predetermined total | 10 | 4 | 1 | 7 | 8 | 25 | 9 | 64 | 8.9 | 2 | 9 | 5 | 0 | 5 | 0 | 4 | 25 | 8.6 | 0 | 6 | 0 | 4 | 2 | 12 | 9.7 | 101 | 8.9 |
| Retouched/Shaped | | | | | | | | | | | | | | | | | | | | | | | | | | | |
| Scraper | 1 | 5 | 1 | 1 | 1 | 11 | 3 | 23 | 32 | 4 | 2 | 1 | | 2 | 1 | 2 | 12 | 4.1 | | 3 | | 1 | | 4 | 32 | 39 | 3.4 |
| End-scraper | | 1 | 2 | 2 | | 4 | | 9 | 13 | 1 | | 2 | | 2 | | 1 | 6 | 2.1 | | 1 | 1 | | 1 | 3 | 2.4 | 18 | 1.6 |
| Truncation | | 2 | | | | 1 | 1 | 4 | 06 | | | 1 | | | | 1 | 2 | 0.7 | | | | 1 | | 1 | 08 | 7 | 06 |
| Beck | 1 | 2 | | | 1 | 1 | | 5 | 0.7 | | 1 | 1 | | 1 | | | 3 | 1.0 | | 3 | | 1 | | 4 | 32 | 12 | 1.1 |
| Denticulate | 5 | 3 | 1 | | 3 | 17 | 3 | 32 | 4.4 | 1 | 7 | 4 | | 5 | 1 | 4 | 22 | 7.6 | 1 | 1 | | 4 | 1 | 7 | 5.6 | 61 | 5.4 |
| Point | | | | | | | | | | 1 | | | | | | 2 | 3 | 1.0 | | | | | 1 | 1 | 08 | 4 | 0.4 |
| Backed piece | | | | | | | | | | 1 | 1 | | | | | | 2 | 0.7 | | | | | | | | 2 | 02 |
| Tanged tool | | | | | | | | | | | | 1 | | | | | 1 | 03 | | | | | 1 | 1 | 08 | 2 | 02 |
| Bifacial tool | | 1 | | | 3 | | | 4 | 06 | | | | | | | | | | | | | | | | | 4 | 0.4 |
| Biface preform | | | | | | | 1 | 1 | 0.1 | | | | | | | | | | | | | | | | | 1 | 0.1 |
| Retouched undeterminable | | 1 | | | 1 | 1 | | 3 | 0.4 | 2 | | | | 1 | | 1 | 4 | 1.4 | 1 | 2 | | 2 | | 5 | 40 | 12 | 1.1 |
| Retouched total | 7 | 15 | 4 | 3 | 9 | 35 | 8 | 81 | 113 | 10 | 11 | 10 | 0 | 11 | 2 | 11 | 55 | 189 | 2 | 10 | 1 | 9 | 4 | 26 | 210 | 162 | 143 |
| Core | | | | | | | | | | | | | | | | | | | | | | | | | | | |
| Core pre-form | | | | | 1 | 2 | | 3 | 0.4 | | 2 | | | | | | 2 | 0.7 | | | | | | | | 5 | 0.4 |
| Core on flake | | | | | | | | | | | | 1 | | | | 1 | 2 | 0.7 | | 1 | | | | 1 | 08 | 3 | 03 |
| Discoid core | | | | | | | | | | | | 1 | | 1 | | | 2 | 0.7 | | | | | | | | 2 | 02 |
| Laminar flakes core | | | | | | | | | | | | 2 | | | | | 2 | 0.7 | | | | | | | | 2 | 02 |
| Levallois core (flakes) | | | | | | 1 | | 1 | 0.1 | | | | | | | 1 | 1 | 03 | | | | | 1 | 1 | 08 | 3 | 03 |
| Levallois core (points) | | | | | | | | | | 1 | | | | | | | 1 | 03 | | | | | | | | 1 | 0.1 |
| Unstructured core | | 1 | | | 1 | 2 | | 4 | 0.6 | 2 | | 3 | | 1 | | 2 | 8 | 2.7 | | 1 | | | 1 | 2 | 1.6 | 14 | 12 |
| Blade core | | | | | | | | | | | | | | 1 | | | 1 | 03 | | | | | | | | 1 | 0.1 |
| Core total | | 1 | | | 2 | 5 | | 8 | 1.1 | 3 | 2 | 7 | | 3 | | 4 | 19 | 6.5 | | 2 | | | 2 | 4 | 32 | 31 | 2.7 |
| Total | 107 | 83 | 31 | 43 | 81 | 306 | 69 | 720 | 100.0 | 35 | 47 | 80 | 10 | 52 | 10 | 57 | 291 | 100.0 | 9 | 50 | 6 | 38 | 21 | 124 | 100.0 | 1135 | 100.0 |

**Table SI 5:** Descriptive statistics for artefacts' metric data (Length, Width, Thickness, in mm) from a subset of samples (n = 241) from sites 16/15, 15/1 and 16/29 (grouped) according to defined groups of degree of surface alteration (See Fig. SI 8)

| Group | | Length | Width | Thickness |
|---|---|---|---|---|
| Group 1 | N | 159 | 159 | 159 |
| | Mean | 42.16 | 35.01 | 12.72 |
| | Median | 35 | 28 | 9 |
| | Std. Deviation | 26.794 | 22.704 | 14.739 |
| | Variance | 717.897 | 515.475 | 217.239 |
| | Minimum | 9 | 7 | 2 |
| | Maximum | 167 | 165 | 135 |
| Group 2 | N | 62 | 62 | 62 |
| | Mean | 34.35 | 29.71 | 10.27 |
| | Median | 29 | 25.5 | 9 |
| | Std. Deviation | 18.16 | 15.774 | 5.823 |
| | Variance | 329.774 | 248.832 | 33.907 |
| | Minimum | 5 | 7 | 2 |
| | Maximum | 90 | 82 | 27 |
| Group 3 | N | 20 | 20 | 20 |
| | Mean | 30.2 | 25.05 | 8.55 |
| | Median | 29 | 24.5 | 7.5 |
| | Std. Deviation | 13.74 | 8.432 | 4.673 |
| | Variance | 188.8 | 71.103 | 21.839 |
| | Minimum | 9 | 11 | 3 |
| | Maximum | 57 | 46 | 22 |
| Total | N | 241 | 241 | 241 |
| | Mean | 39.16 | 32.82 | 11.75 |
| | Median | 34 | 27 | 9 |
| | Std. Deviation | 24.29 | 20.468 | 12.466 |
| | Variance | 590.011 | 418.925 | 155.407 |
| | Minimum | 5 | 7 | 2 |
| | Maximum | 167 | 165 | 135 |

**Table SI 6**: a - mean values of core Length, Width, Thickness (in mm) grouped per type of core and site (unpatterned cores and pre-forms not considered); b - individual core Length, Width, Thickness values.

a

| Site | Core type | Mean L | Mean W | Mean T |
|---|---|---|---|---|
| 16_29 | Levallois (flakes) | 142.0 | 93.0 | 33.0 |
| | On flake | 52.0 | 85.0 | 15.0 |
| 15_1 | Levallois (flakes) | 51.0 | 47.0 | 19.0 |
| | Levallois (points) | 57.0 | 63.0 | 34.0 |
| | Laminar | 150.0 | 91.0 | 84.0 |
| | Laminar flakes | 154.5 | 119.5 | 99.5 |
| | Discoid | 38.5 | 37.0 | 20.0 |
| | On flake | 49.5 | 39.0 | 24.0 |
| 16_15 | Levallois (flakes) | 117.0 | 73.0 | 28.0 |

b

| Site | Layer | Core type | L | W | T | Type of cortex | Integrity | ID |
|---|---|---|---|---|---|---|---|---|
| 16_29 | 4 | On flake | 52 | 85 | 15 | Nodule | Whole | 187 |
| | 7 | Levallois (flakes) | 142 | 93 | 33 | Nodule | Whole | 1136 |
| 15_1 | 4a | Levallois (points) | 57 | 63 | 34 | Nodule | Whole | 910 |
| | 5 | Discoid | 36 | 39 | 22 | Not cortical | Whole | 980 |
| | 5 | Laminar flakes | 167 | 165 | 135 | Nodule | Whole | 242 |
| | 5 | Laminar flakes | 142 | 74 | 64 | Nodule | Whole | 43 |
| | 5 | On flake | 63 | 45 | 30 | Not cortical | Whole | 998 |
| | 7 | Laminar | 150 | 91 | 84 | Nodule | Whole | 83 |
| | 7 | Discoid | 41 | 35 | 18 | Nodule | Whole | 73 |
| | 9-10 | Levallois (flakes) | 51 | 47 | 19 | Nodule | Whole | 1079 |
| | 9-10 | On flake | 36 | 33 | 18 | Nodule | Whole | 1052 |
| 16_15 | I | Levallois (flakes) | 117 | 73 | 28 | Nodule | Whole | 307 |

**Table SI 7**: Dorsal scars pattern on Levallois flakes (retouched and unretouched) according to site and layer

| | | **Dorsal scars pattern** | | | | | | | | | | | |
|---|---|---|---|---|---|---|---|---|---|---|---|---|---|
| **Site** | **Layer** | **Unidirectional** | | **Bidirectioal** | | **Centripetal** | | **Convergent** | | **Orthogonal** | | **Total** | |
| | | **n** | **%** | **n** | **%** | **n** | **%** | **n** | **%** | **n** | **%** | **n** | **%** |
| **16_29** | 4 | | | | | 1 | 50.0% | 1 | 50.0% | | | 2 | 100,0% |
| | 6 | | | | | | | 1 | 50.0% | 1 | 50.0% | 2 | 100,0% |
| | 7 | | | | | 2 | 100.0% | | | | | 2 | 100,0% |
| | **16_29 Total** | | | | | **3** | **50.0%** | **2** | **33.3%** | **1** | **16.7%** | **6** | **100,0%** |
| **15_1** | 4a | 1 | 33.3% | | | 1 | 33.3% | | | 1 | 33.3% | 3 | 100,0% |
| | 4b | 1 | 50.0% | 1 | 50.0% | | | | | | | 2 | 100,0% |
| | 5 | 1 | 50.0% | | | 1 | 50.0% | | | | | 2 | 100,0% |
| | 7 | 1 | 50.0% | | | | | 1 | 50.0% | | | 2 | 100,0% |
| | 9-10 | 2 | 50.0% | 1 | 25.0% | | | | | 1 | 25.0% | 4 | 100,0% |
| | **15_1 Total** | **6** | **46.2%** | **2** | **15.4%** | **2** | **15.4%** | **1** | **7.7%** | **2** | **15.4%** | **13** | **100,0%** |
| **16_15** | A | 4 | 57.1% | 1 | 14.3% | 2 | 28.6% | | | | | 7 | 100,0% |
| | BC | | | | | 5 | 83.3% | | | 1 | 16.7% | 6 | 100,0% |
| | DEF | 2 | 66.7% | | | 1 | 33.3% | | | | | 3 | 100,0% |
| | G | 1 | 33.3% | | | 1 | 33.3% | 1 | 33.3% | | | 3 | 100,0% |
| | H | 3 | 42.9% | 2 | 28.6% | | | 2 | 28.6% | | | 7 | 100,0% |
| | I | 9 | 37.5% | 1 | 4.2% | 10 | 41.7% | 2 | 8.3% | 2 | 8.3% | 24 | 100,0% |
| | J | | | 1 | 12.5% | 4 | 50.0% | 2 | 25.0% | 1 | 12.5% | 8 | 100,0% |
| | **16_15 Total** | **19** | **32.8%** | **5** | **8.6%** | **23** | **39.7%** | **7** | **12.1%** | **4** | **6.9%** | **58** | **100,0%** |
| **Total** | | **25** | **32,5%** | **7** | **9.1%** | **28** | **36.4%** | **10** | **13.0%** | **7** | **9.1%** | **77** | **100.0%** |

**Table SI 8:** Recognizable platform of Levallois flakes (retouched and unretouched) according to site and layer

| | | Platform | | | | | | | |
|---|---|---|---|---|---|---|---|---|---|
| | | **Facetted** | | **Dihedral** | | **Plain** | | **Total** | |
| **Site** | **Layer** | **n** | **%** | **n** | **%** | **n** | **%** | **n** | **%** |
| **16_29** | 4 | 1 | 50.0% | 1 | 50.0% | | | 2 | 100,0% |
| | 6 | 1 | 100.0% | | | | | 1 | 100,0% |
| | 7 | 2 | 100.0% | | | | | 2 | 100,0% |
| | **16_29 Total** | **4** | **80.0%** | **1** | **20.0%** | | | **5** | **100,0%** |
| **15_1** | 4a | 1 | 50.0% | | | 1 | 50.0% | 2 | 100,0% |
| | 4b | 1 | 50.0% | 1 | 50.0% | | | 2 | 100,0% |
| | 5 | 1 | 50.0% | | | 1 | 50.0% | 2 | 100,0% |
| | 7 | 1 | 50.0% | | | 1 | 50.0% | 2 | 100,0% |
| | 9-10 | 2 | 50.0% | | | 2 | 50.0% | 4 | 100,0% |
| | **15_1 Total** | **6** | **50.0%** | **1** | **8.3%** | **5** | **41.7%** | **12** | **100,0%** |
| **16_15** | A | 3 | 60.0% | 1 | 20.0% | 1 | 20.0% | 5 | 100,0% |
| | BC | 3 | 50.0% | 1 | 16.7% | 2 | 33.3% | 6 | 100,0% |
| | G | 2 | 66.7% | | | 1 | 33.3% | 3 | 100,0% |
| | H | 2 | 50.0% | 1 | 25.0% | 1 | 25.0% | 4 | 100,0% |
| | I | 9 | 50.0% | 2 | 11.1% | 7 | 38.9% | 18 | 100,0% |
| | J | 3 | 50.0% | 1 | 16.7% | 2 | 33.3% | 6 | 100,0% |
| | **16_15 Total** | **22** | **52.4%** | **6** | **14.3%** | **14** | **33.3%** | **42** | **100,0%** |
| **Total** | | **32** | **54,2%** | **8** | **13.6%** | **19** | **32.2%** | **59** | **100.0%** |

**Table SI 9:** Summary statistics for Lenght, Width, Thickness (in mm) of whole Levallois flakes (n = 54) from sites 16/15, 15/1 and 16/29

**Site 16/15**

| Layer | | Length | Width | Thickness |
|---|---|---|---|---|
| A (n = 6) | N | 6 | 6 | 6 |
| | Mean | 67.5 | 51.83 | 12.33 |
| | Minimum | 40 | 31 | 9 |
| | Maximum | 102 | 80 | 16 |
| | Std. Deviation | 21.742 | 18.433 | 2.503 |
| BC (n = 6) | N | 6 | 6 | 6 |
| | Mean | 58.17 | 41.17 | 11.5 |
| | Minimum | 29 | 26 | 7 |
| | Maximum | 86 | 63 | 15 |
| | Std. Deviation | 20.537 | 15.012 | 3.782 |
| DEF (n =2) | N | 2 | 2 | 2 |
| | Mean | 49 | 45.5 | 10.5 |
| | Minimum | 31 | 37 | 7 |
| | Maximum | 67 | 54 | 14 |
| G (n=2) | N | 2 | 2 | 2 |
| | Mean | 44 | 42.5 | 8.5 |
| | Minimum | 40 | 37 | 7 |
| | Maximum | 48 | 48 | 10 |
| H (n=7) | N | 7 | 7 | 7 |
| | Mean | 48.14 | 40.43 | 11.14 |
| | Minimum | 32 | 28 | 7 |
| | Maximum | 79 | 58 | 17 |
| | Std. Deviation | 18.497 | 11.487 | 3.848 |
| I (n=12) | N | 12 | 12 | 12 |
| | Mean | 61.42 | 42 | 12.67 |
| | Minimum | 27 | 12 | 6 |
| | Maximum | 99 | 63 | 19 |
| | Std. Deviation | 21.861 | 15.661 | 3.42 |
| J (n=6) | N | 6 | 6 | 6 |
| | Mean | 61.33 | 42.67 | 9.83 |
| | Minimum | 33 | 29 | 7 |
| | Maximum | 75 | 71 | 12 |
| | Std. Deviation | 18.096 | 14.787 | 2.483 |
| Total (n=41) | N | 41 | 41 | 41 |
| | Mean | 58.1 | 43.34 | 11.46 |
| | Minimum | 27 | 12 | 6 |
| | Maximum | 102 | 80 | 19 |
| | Std. Deviation | 20.051 | 14.291 | 3.31 |

**Site 15/1**

| Layer | | Length | Width | Thickness |
|---|---|---|---|---|
| 4a (n = 3) | Mean | 56 | 41 | 12.33 |
| | Minimum | 43 | 29 | 8 |
| | Maximum | 71 | 62 | 19 |
| | Std. Deviation | 14.107 | 18.248 | 5.859 |
| 4b (n =1) | | 44 | 24 | 6 |
| 5 (n = 2) | Mean | 55.5 | 45 | 13 |
| | Minimum | 52 | 36 | 12 |
| | Maximum | 59 | 54 | 14 |
| 7 (n=1) | | 43 | 29 | 11 |
| 9_10 (n=3) | Mean | 45 | 28 | 6.67 |
| | Minimum | 31 | 13 | 4 |
| | Maximum | 53 | 39 | 9 |
| | Std. Deviation | 12.166 | 13.454 | 2.517 |
| Total (n=10) | Mean | 50.1 | 35 | 10 |
| | Minimum | 31 | 13 | 4 |
| | Maximum | 71 | 62 | 19 |
| | Std. Deviation | 10.785 | 14.15 | 4.32 |

**Site 16/29**

| Layer | | Length | Width | Thickness |
|---|---|---|---|---|
| 6 (n = 1) | | 79 | 43 | 12 |
| 7 (n =2) | Mean | 29.5 | 34 | 6.5 |
| | Minimum | 27 | 31 | 6 |
| | Maximum | 32 | 37 | 7 |
| | Std. Deviation | 3.536 | 4.243 | 0.707 |
| Total (n = 3) | Mean | 46 | 37 | 8.33 |
| | Minimum | 27 | 31 | 6 |
| | Maximum | 79 | 43 | 12 |
| | Std. Deviation | 28.688 | 6 | 3.215 |

**All sites**

| | | | | |
|---|---|---|---|---|
| Total (n = 54) | Mean | 55.94 | 41.44 | 11.02 |
| | Minimum | 27 | 12 | 4 |
| | Maximum | 102 | 80 | 19 |
| | Std. Deviation | 19.232 | 14.186 | 3.547 |

**Table SI 10**: Dorsal scars pattern on blades (retouched and unretouched) according to site and layer

| | | **Bidirectional** | | **Unidirectional** | | **Orthogonal** | | **Total** | |
|---|---|---|---|---|---|---|---|---|---|
| **Site** | **Layer** | **n** | **%** | **n** | **%** | **n** | **%** | **n** | **%** |
| **16_29** | 4 | | | 3 | 100.0% | | | 3 | 100.0% |
| | 7 | | | 1 | 100.0% | | | 1 | 100.0% |
| | **16_29 Total** | | | **4** | **100.0%** | | | **4** | **100.0%** |
| **15_1** | 4a | | | 2 | 66.7% | 1 | 33.3% | 3 | 100.0% |
| | 4b | | | 2 | 100.0% | | | 2 | 100.0% |
| | 5 | 1 | 14.3% | 6 | 85.7% | | | 7 | 100.0% |
| | 7 | 1 | 25.0% | 2 | 50.0% | 1 | 25.0% | 4 | 100.0% |
| | 9-10 | | | 2 | 100.0% | | | 2 | 100.0% |
| | **15_1 Total** | **2** | **11.1%** | **14** | **77.8%** | **2** | **11.1%** | **18** | **100.0%** |
| **16_15** | A | | | 5 | 100.0% | | | 5 | 100.0% |
| | BC | | | | | 1 | 100.0% | 1 | 100.0% |
| | G | | | 3 | 100.0% | | | 3 | 100.0% |
| | H | | | 3 | 100.0% | | | 3 | 100.0% |
| | I | | | 6 | 85.7% | 1 | 14.3% | 7 | 100.0% |
| | J | | | 1 | 50.0% | 1 | 50.0% | 2 | 100.0% |
| | **16_15 Total** | | | **18** | **85.7%** | **3** | **14.3%** | **21** | **100.0%** |
| **Total** | | **2** | **4.7%** | **36** | **83.7%** | **5** | **11.6%** | **43** | **100.0%** |

**Tab. SI 11**: Recognizable platform of blades (retouched and unretouched) according to site and layer

| Site | | Cortical | | Facetted | | Plain | | Punctiform | | Undeterminable | | Total | |
|---|---|---|---|---|---|---|---|---|---|---|---|---|---|
| | **Layer** | **n** | **%** | **n** | **%** | **n** | **%** | **n** | **%** | **n** | **%** | **n** | **%** |
| **16_29** | 4 | | | 1 | 100.0% | | | | | | | 1 | 100.0% |
| | **16_29 Total** | | | **1** | **100.0%** | | | | | | | **1** | **100.0%** |
| **15_1** | 4a | | | 1 | 50.0% | 1 | 50.0% | | | | | 2 | 100.0% |
| | 4b | | | 1 | 50.0% | | | 1 | 50.0% | | | 2 | 100.0% |
| | 5 | 1 | 20.0% | 1 | 20.0% | 3 | 60.0% | | | | | 5 | 100.0% |
| | 7 | | | 1 | 25.0% | 2 | 50.0% | | | 1 | 25.0% | 4 | 100.0% |
| | **15_1 Total** | **1** | **7.7%** | **4** | **30.8%** | **6** | **46.2%** | **1** | **7.7%** | **1** | **7.7%** | **13** | **100.0%** |
| **16_15** | A | | | | | 2 | 100.0% | | | | | 2 | 100.0% |
| | BC | | | | | 1 | 100.0% | | | | | 1 | 100.0% |
| | G | | | | | 1 | 100.0% | | | | | 1 | 100.0% |
| | H | | | | | 1 | 100.0% | | | | | 1 | 100.0% |
| | I | 1 | 20.0% | | | 3 | 60.0% | | | 1 | 20.0% | 5 | 100.0% |
| | J | | | | | | | | | 1 | 100.0% | 1 | 100.0% |
| | **16_15 Total** | **1** | **9.1%** | | | **8** | **72.7%** | | | **2** | **18.2%** | **11** | **100.0%** |
| **Total** | | **2** | **8.0%** | **5** | **20.0%** | **14** | **56.0%** | **1** | **4.0%** | **3** | **12.0%** | **25** | **100.0%** |

**Table SI 12**: Summary statistics for Length, Width, Thickness (in mm) of whole blades (n = 20) from sites 16/15, 15/1 and 16/29.

| Site | | L | W | T |
|---|---|---|---|---|
| 15_1 | N | 11 | 11 | 11 |
| | Mean | 52.09 | 23 | 9.82 |
| | Minimum | 31 | 14 | 3 |
| | Maximum | 71 | 33 | 15 |
| | Std. Deviation | 13.831 | 5.514 | 3.188 |
| 16_15 | N | 9 | 9 | 9 |
| | Mean | 53 | 25.78 | 11.56 |
| | Minimum | 28 | 14 | 4 |
| | Maximum | 82 | 43 | 20 |
| | Std. Deviation | 19.887 | 9.338 | 5.457 |
| Total | N | 20 | 20 | 20 |
| | Mean | 52.5 | 24.25 | 10.6 |
| | Minimum | 28 | 14 | 3 |
| | Maximum | 82 | 43 | 20 |
| | Std. Deviation | 16.353 | 7.398 | 4.321 |

**Table SI 13**: Summary statistics for Length, Width, Thickness (in mm) of whole Discoid flakes (n = 6) and Kombewa flakes (n = 4) from sites 16/15, 15/1 and 16/29.

Discoid flake

| Site | | L | W | T |
|---|---|---|---|---|
| 16_29 | N | 2 | 2 | 2 |
| | Mean | 42.5 | 50.5 | 13 |
| | Minimum | 37 | 32 | 7 |
| | Maximum | 48 | 69 | 19 |
| | Std. Deviation | 7.778 | 26.163 | 8.485 |
| 15_1 | N | 3 | 3 | 3 |
| | Mean | 38 | 37.33 | 13 |
| | Minimum | 32 | 29 | 12 |
| | Maximum | 47 | 43 | 14 |
| | Std. Deviation | 7.937 | 7.371 | 1 |
| 16_15 | N | 1 | 1 | 1 |
| | | 35 | 37 | 7 |
| | Minimum | . | . | . |
| | Maximum | . | . | . |
| | Std. Deviation | . | . | . |
| Total | N | 6 | 6 | 6 |
| | Mean | 39 | 41.67 | 12 |
| | Minimum | 32 | 29 | 7 |
| | Maximum | 48 | 69 | 19 |
| | Std. Deviation | 6.782 | 14.334 | 4.561 |

Kombewa flake

| Site | | L | W | T |
|---|---|---|---|---|
| 16_29 | N | 1 | 1 | 1 |
| | | 30 | 27 | 9 |
| | Minimum | . | . | . |
| | Maximum | . | . | . |
| | Std. Deviation | . | . | . |
| 15_1 | N | 1 | 1 | 1 |
| | | 33 | 30 | 5 |
| | Minimum | . | . | . |
| | Maximum | . | . | . |
| | Std. Deviation | . | . | . |
| 16_15 | N | 2 | 2 | 2 |
| | Mean | 31 | 49.5 | 14.5 |
| | Minimum | 30 | 45 | 12 |
| | Maximum | 32 | 54 | 17 |
| | Std. Deviation | 1.414 | 6.364 | 3.536 |
| Total | N | 4 | 4 | 4 |
| | Mean | 31.25 | 39 | 10.75 |
| | Minimum | 30 | 27 | 5 |
| | Maximum | 33 | 54 | 17 |
| | Std. Deviation | 1.5 | 12.728 | 5.058 |

**Table SI 14:** Summary statistics for Lenght, Width, Thickness (in mm) of whole retouched blanks (n =89) from sites 16/15, 15/1 and 16/29. Tool types considered are (grouped): scraper, end-scraper, denticulate, notch, beck, point, truncation

16_15

| Layer | | L | W | T |
|---|---|---|---|---|
| A | N | 6 | 6 | 6 |
| | Mean | 53.17 | 52.67 | 14.17 |
| | Min | 30 | 22 | 8 |
| | Max | 80 | 91 | 27 |
| | Std. Dev. | 18.691 | 28.296 | 6.969 |
| BC | N | 11 | 11 | 11 |
| | Mean | 39.82 | 34.09 | 9.27 |
| | Min | 24 | 17 | 4 |
| | Max | 66 | 70 | 15 |
| | Std. Dev. | 13.519 | 16.003 | 3.409 |
| DEF | N | 3 | 3 | 3 |
| | Mean | 35.67 | 34 | 8 |
| | Min | 31 | 28 | 7 |
| | Max | 40 | 37 | 10 |
| | Std. Dev. | 4.509 | 5.196 | 1.732 |
| G | N | 3 | 3 | 3 |
| | Mean | 71.33 | 62 | 16.33 |
| | Min | 61 | 52 | 10 |
| | Max | 83 | 77 | 21 |
| | Std. Dev. | 11.06 | 13.229 | 5.686 |
| H | N | 2 | 2 | 2 |
| | Mean | 40 | 36 | 7.5 |
| | Min | 37 | 32 | 7 |
| | Max | 43 | 40 | 8 |
| | Std. Dev. | 4.243 | 5.657 | 0.707 |
| I | N | 18 | 18 | 18 |
| | Mean | 70.56 | 49.39 | 15.11 |
| | Min | 17 | 12 | 6 |
| | Max | 154 | 165 | 32 |
| | Std. Dev. | 39.364 | 33.051 | 7.012 |
| J | N | 4 | 4 | 4 |
| | Mean | 51.25 | 31.5 | 10.75 |
| | Min | 31 | 28 | 7 |
| | Max | 71 | 35 | 17 |
| | Std. Dev. | 17.746 | 2.887 | 4.787 |
| Total | N | 47 | 47 | 47 |
| | Mean | 56.02 | 43.96 | 12.55 |
| | Min | 17 | 12 | 4 |
| | Max | 154 | 165 | 32 |
| | Std. Dev. | 29.656 | 25.399 | 6.178 |

15_1

| Layer | | L | W | T |
|---|---|---|---|---|
| 4a | N | 6 | 6 | 6 |
| | Mean | 53.33 | 34.67 | 13 |
| | Min | 37 | 23 | 5 |
| | Max | 71 | 62 | 19 |
| | Std. Dev. | 15.475 | 13.924 | 5.367 |
| 4b | N | 7 | 7 | 7 |
| | Mean | 45.29 | 39 | 13 |
| | Min | 24 | 24 | 6 |
| | Max | 64 | 71 | 21 |
| | Std. Dev. | 13.961 | 18.157 | 5.416 |
| 5 | N | 8 | 8 | 8 |
| | Mean | 43.75 | 26.25 | 10.38 |
| | Min | 30 | 14 | 7 |
| | Max | 60 | 36 | 13 |
| | Std. Dev. | 12.407 | 7.402 | 1.996 |
| 7 | N | 5 | 5 | 5 |
| | Mean | 31 | 42.4 | 17 |
| | Min | 12 | 16 | 8 |
| | Max | 43 | 94 | 43 |
| | Std. Dev. | 13.435 | 30.386 | 14.612 |
| 9_10 | N | 5 | 5 | 5 |
| | Mean | 46.6 | 27.8 | 8.6 |
| | Min | 24 | 13 | 4 |
| | Max | 81 | 52 | 16 |
| | Std. Dev. | 22.278 | 15.139 | 4.506 |
| Total | N | 31 | 31 | 31 |
| | Mean | 44.35 | 33.61 | 12.26 |
| | Min | 12 | 13 | 4 |
| | Max | 81 | 94 | 43 |
| | Std. Dev. | 15.876 | 17.454 | 7.066 |

16_29

| Layer | | L | W | T |
|---|---|---|---|---|
| 3 | N | 1 | 1 | 1 |
| | Mean | 92 | 77 | 32 |
| | Min | 92 | 77 | 32 |
| | Max | 92 | 77 | 32 |
| | Std. Dev. | . | . | . |
| 4 | N | 4 | 4 | 4 |
| | Mean | 57.25 | 59.25 | 15.5 |
| | Min | 33 | 25 | 5 |
| | Max | 90 | 82 | 27 |
| | Std. Dev. | 26.75 | 24.541 | 10.661 |
| 5 | N | 1 | 1 | 1 |
| | Mean | 23 | 38 | 8 |
| | Min | 23 | 38 | 8 |
| | Max | 23 | 38 | 8 |
| | Std. Dev. | . | . | . |
| 6 | N | 4 | 4 | 4 |
| | Mean | 39.75 | 28.25 | 14.75 |
| | Min | 19 | 17 | 7 |
| | Max | 63 | 47 | 31 |
| | Std. Dev. | 19.311 | 13.15 | 11.026 |
| 7 | N | 1 | 1 | 1 |
| | Mean | 27 | 31 | 6 |
| | Min | 27 | 31 | 6 |
| | Max | 27 | 31 | 6 |
| | Std. Dev. | . | . | . |
| Total | N | 11 | 11 | 11 |
| | Mean | 48.18 | 45.09 | 15.18 |
| | Minimum | 19 | 17 | 5 |
| | Maximum | 92 | 82 | 32 |
| | Std. Deviation | 26.233 | 23.514 | 10.61 |

All sites

| | | | | |
|---|---|---|---|---|
| Total | N | 89 | 89 | 89 |
| | Mean | 50.99 | 40.49 | 12.78 |
| | Min | 12 | 12 | 4 |
| | Max | 154 | 165 | 43 |
| | Std. Dev. | 25.571 | 23.013 | 7.114 |

**Table SI 15**: Retouched tools' blank according, to site and layer. Tool types considered are (grouped): scraper, end-scraper, denticulate, notch, beck, point, truncation

| | | **Tool blank** | | | | | | | | | | | | | | | |
|---|---|---|---|---|---|---|---|---|---|---|---|---|---|---|---|---|---|
| | | Flake | | Kombewa flake | | Levallois flake | | Management flake (surface) | | Management flake (platform) | | Blade | | Laminar flake | | Total | |
| **Site** | **Layer** | **n** | **%** | **n** | **%** | **n** | **%** | **n** | **%** | **n** | **%** | **n** | **%** | **n** | **%** | **n** | **%** |
| **16_29** | 3 | 1 | *100.0%* | | | | | | | | | | | | | 1 | *100.0%* |
| | 4 | 6 | *75.0%* | | | 1 | *12.5%* | | | 1 | *12.5%* | | | | | 8 | *100.0%* |
| | 5 | 1 | *100.0%* | | | | | | | | | | | | | 1 | *100.0%* |
| | 6 | 5 | *71.4%* | 1 | *14.3%* | 1 | *14.3%* | | | | | | | | | 7 | *100.0%* |
| | 7 | 2 | *66.7%* | | | 1 | *33.3%* | | | | | | | | | 3 | *100.0%* |
| | **16_29 Total** | **15** | ***75.0%*** | **1** | ***5.0%*** | **3** | ***15.0%*** | | | **1** | ***5.0%*** | | | | | **20** | ***100.0%*** |
| **15_1** | 4a | 3 | *42.9%* | | | 2 | *28.6%* | | | | | 2 | *28.6%* | | | 7 | *100.0%* |
| | 4b | 6 | *60.0%* | | | 2 | *20.0%* | 1 | *10.0%* | | | | | 1 | *10.0%* | 10 | *100.0%* |
| | 5 | 4 | *44.4%* | | | 1 | *11.1%* | | | | | 3 | *33.3%* | 1 | *11.1%* | 9 | *100.0%* |
| | 7 | 5 | *50.0%* | 1 | *10.0%* | 1 | *10.0%* | | | | | 2 | *20.0%* | 1 | *10.0%* | 10 | *100.0%* |
| | 8 | 2 | *100.0%* | | | | | | | | | | | | | 2 | *100.0%* |
| | 9-10 | 6 | *60.0%* | | | 2 | *20.0%* | | | 1 | *10.0%* | 1 | *10.0%* | | | 10 | *100.0%* |
| | **15_1 Total** | **26** | ***54.2%*** | **1** | ***2.1%*** | **8** | ***16.7%*** | **1** | ***2.1%*** | **1** | ***2.1%*** | **8** | ***16.7%*** | **3** | ***6.3%*** | **48** | ***100.0%*** |
| **16_15** | A | 4 | *57.1%* | | | 3 | *42.9%* | | | | | | | | | 7 | *100.0%* |
| | BC | 11 | *78.6%* | | | 2 | *14.3%* | | | | | 1 | *7.1%* | | | 14 | *100.0%* |
| | DEF | 2 | *50.0%* | | | 2 | *50.0%* | | | | | | | | | 4 | *100.0%* |
| | G | 3 | *100.0%* | | | | | | | | | | | | | 3 | *100.0%* |
| | H | 2 | *40.0%* | | | 1 | *20.0%* | | | | | 2 | *40.0%* | | | 5 | *100.0%* |
| | I | 22 | *64.7%* | | | 9 | *26.5%* | | | | | 3 | *8.8%* | | | 34 | *100.0%* |
| | J | 6 | *85.7%* | | | 1 | *14.3%* | | | | | | | | | 7 | *100.0%* |
| | **16_15 Total** | **50** | ***67.6%*** | | | **18** | ***24.3%*** | | | | | **6** | ***8.1%*** | | | **74** | ***100.0%*** |
| Total | | 91 | *64.1%* | 2 | *1.4%* | 29 | *20.4%* | 1 | *0.7%* | 2 | *1.4%* | 14 | *9.9%* | 3 | *2.1%* | 142 | *100.0%* |

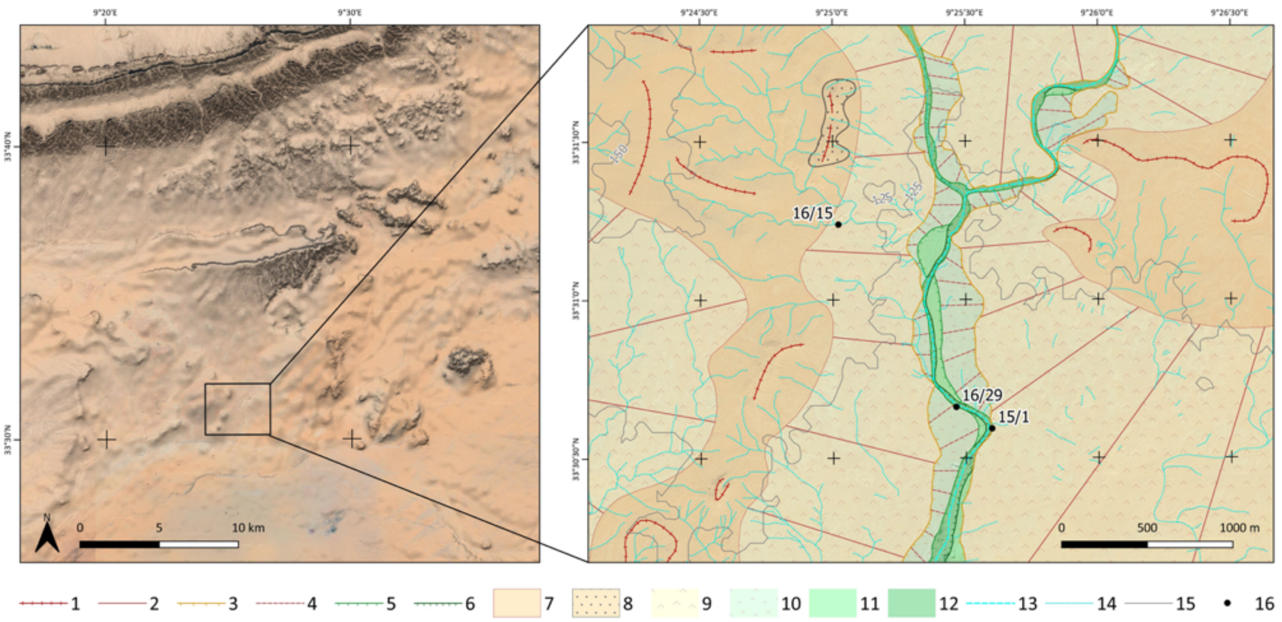


**Fig. SI 1:** Geomorphological sketch of the Wadi Lazalim area (maps created using QGIS 3.20.2 https://qgis.org/it/site/); on the left, the area in its general context (Google Earth satellite imagery with superimposed hillshade model from 30m DTM). 1- Outcrop ridge (limestone); 2- Extension of the erosion glacis 1 and 2; 3- Limit of the erosion glacis 1 and 2; 4- Extension of the piedmont alluvium; 5- Limit of the piedmont alluvium; 6- Holocene terrace; 7- Santonian-Coniacian and Lower Campanian limestone contains flint nodules; 8- Important concentration of flint at the summit of a hill; 9- Gypsum duricrust 80 to 100 cm sealing geological strata and heterometric conglomerate (Mid- to Upper Pleistocene?); 10- Gypsum duricrust 10 to 20 cm thick sealing coarse deposits (Upper Pleistocene?; 11- Coarse deposits with fine matrix (Holocene); 12- Present fluvial deposits; 13- Main Wadi; 14- Wadi; 15- Contour line (25m); 16- Excavated site.

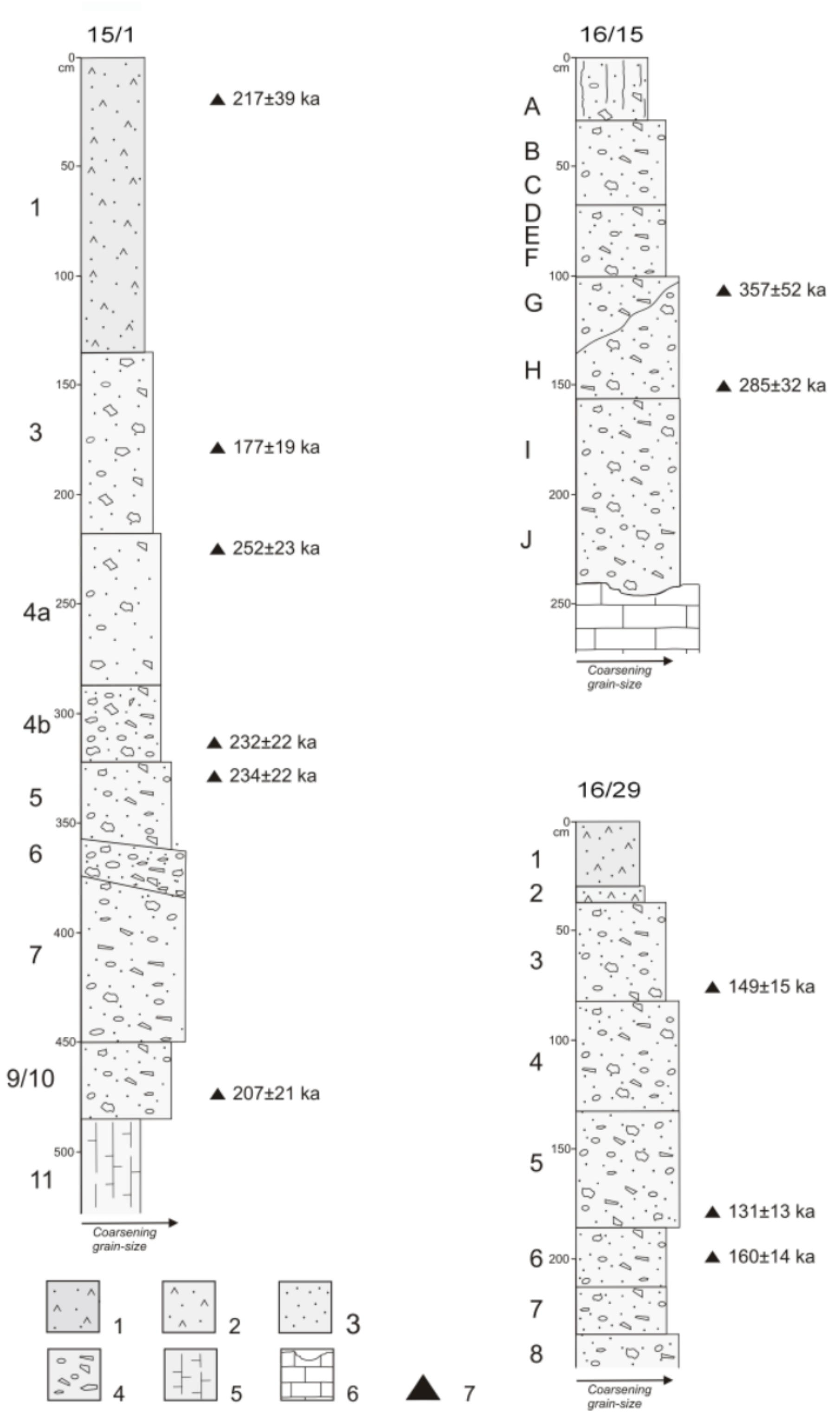


**Fig SI 2:** Interpretative logs of the three investigated sequences. Key: 1) gypsum crust; 2) gypsum crystals in a loose sandy matrix; 3) sandy to silty matrix; 4) coarse constituents; 5) silty to clay deposit; 6) bedrock; 7) position of dated samples. In 16/15, homogeneous units are associated.

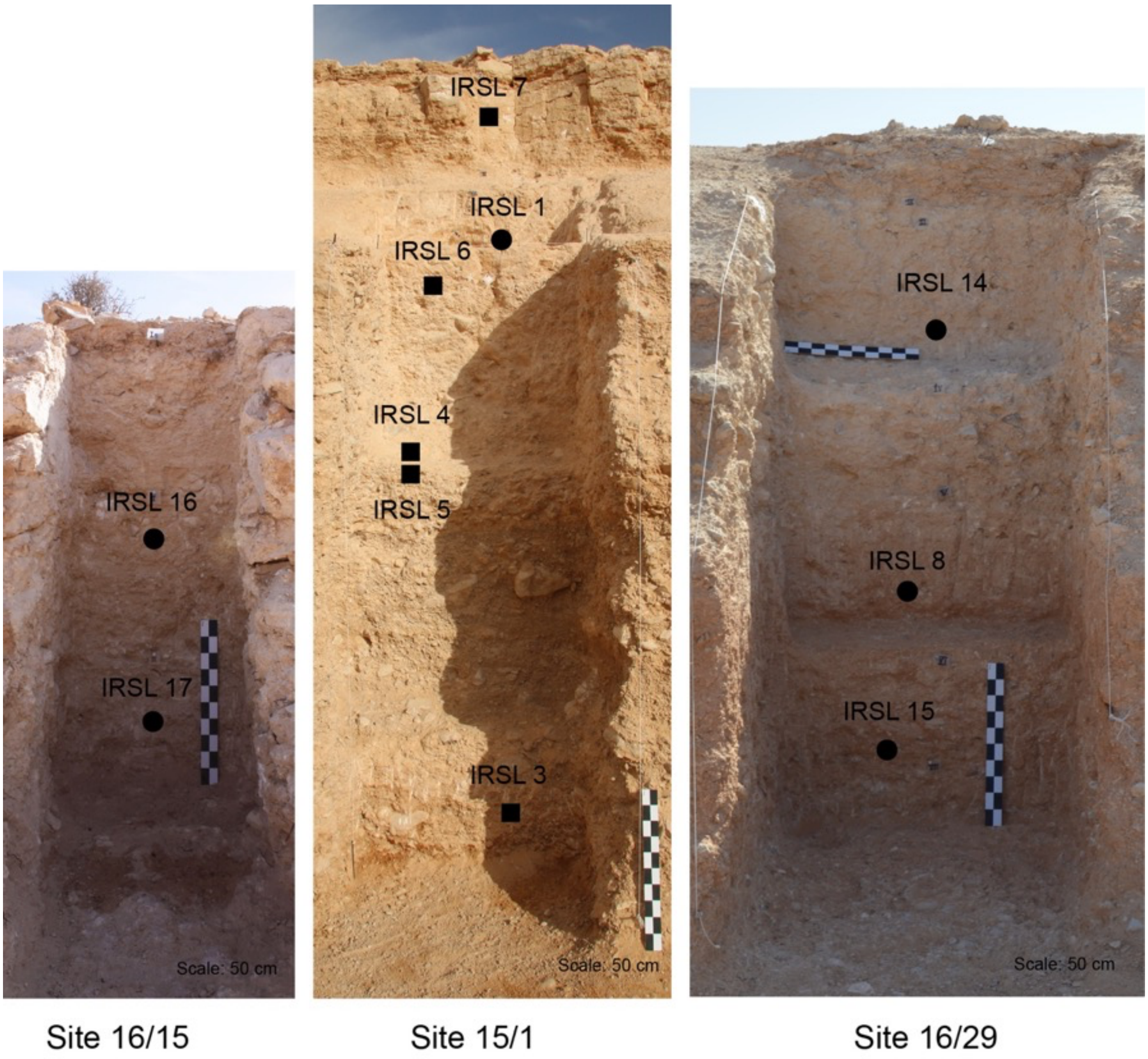


**Fig. SI 3:** Position of samples for IRSL dating collected at sites 16/15, 15/1 and 16/29 on the profiles exposed during the 2016 field mission (scale: 50 cm); circles are metal pipe samples, squares represent sediment blocks.

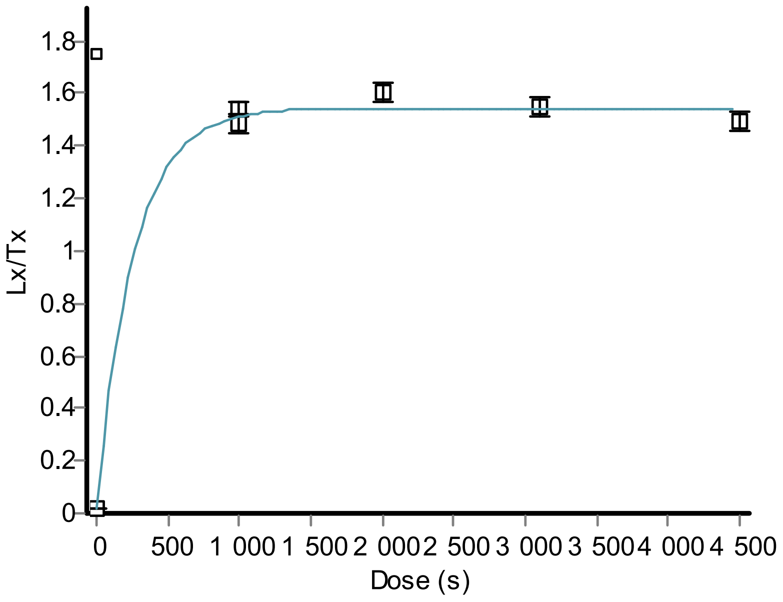


**Fig. SI 4:** BDX 19045 (i.e. IRSL 17). Dose response curve obtained on a quartz multigrain aliquot.

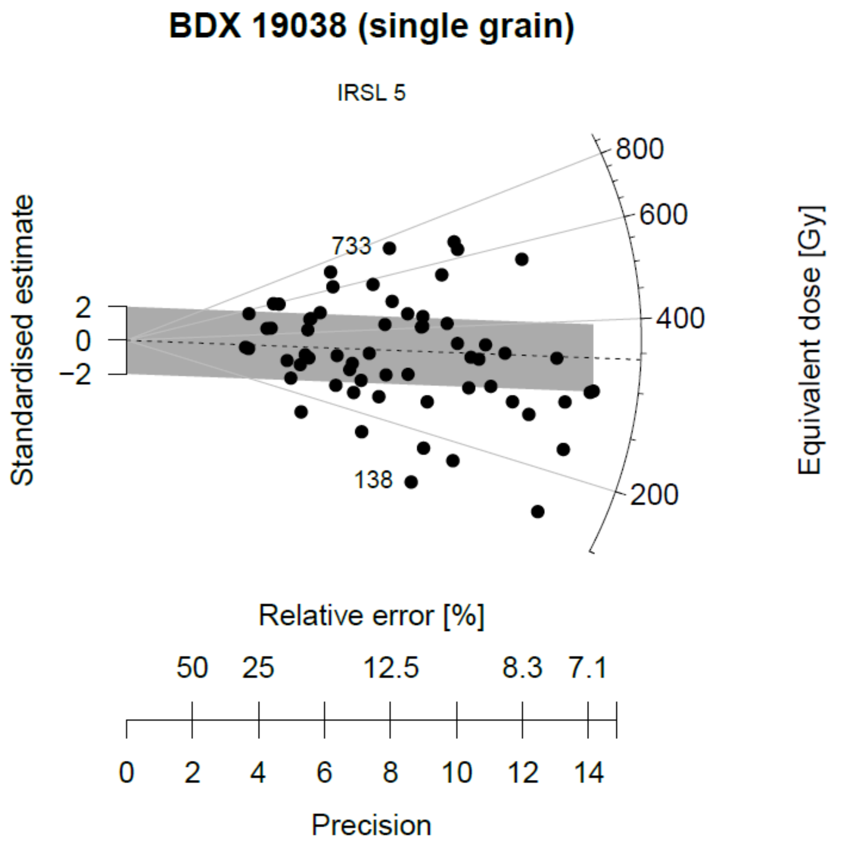


**Fig. SI 5:** BDX 19038 (i.e. IRSL 5). Radial plot showing $pIRIR_{290}$ equivalent doses obtained on single-grain. The post-bleaching residual dose was subtracted (see text).

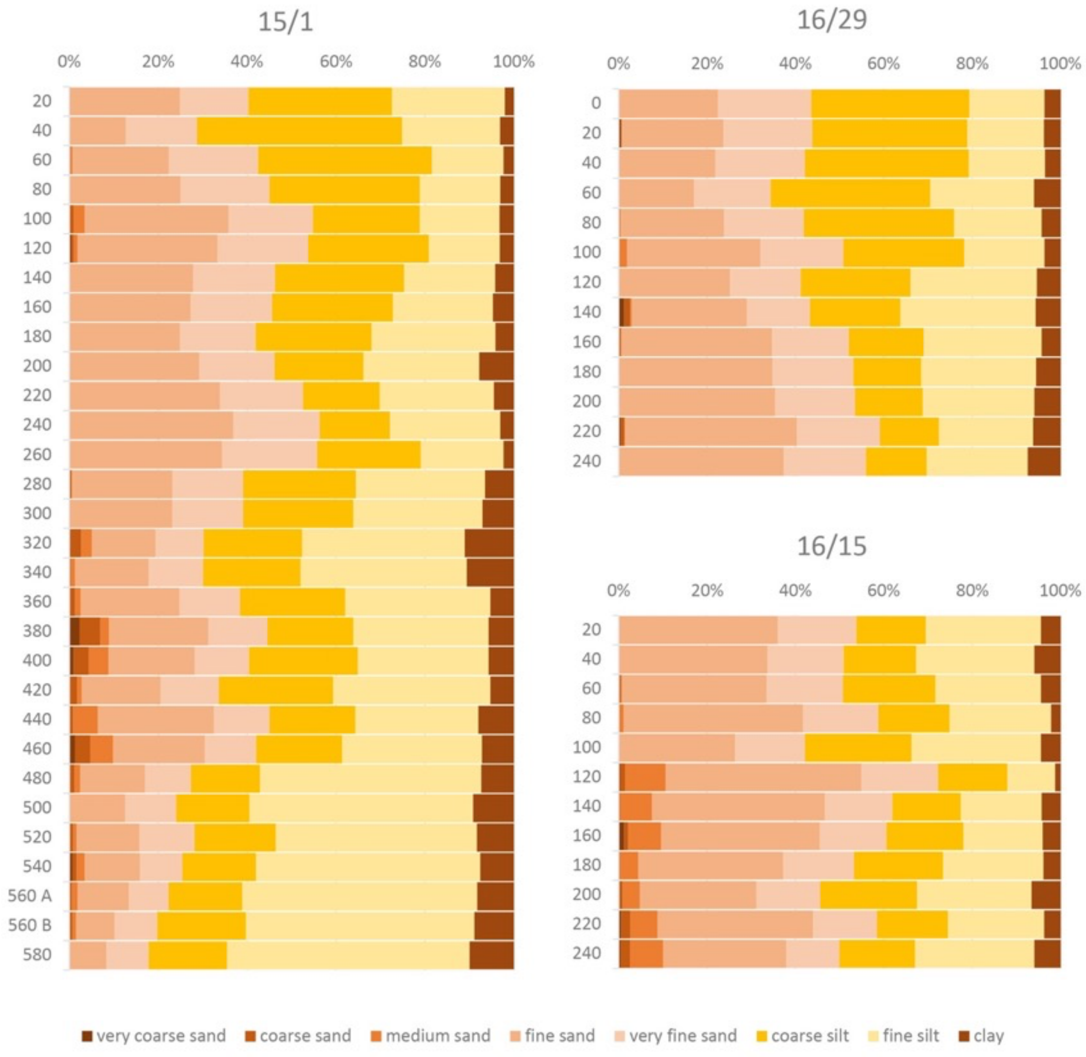


**Fig. SI 6:** Results of grain size analyses on the fine fraction of each investigated sequence.

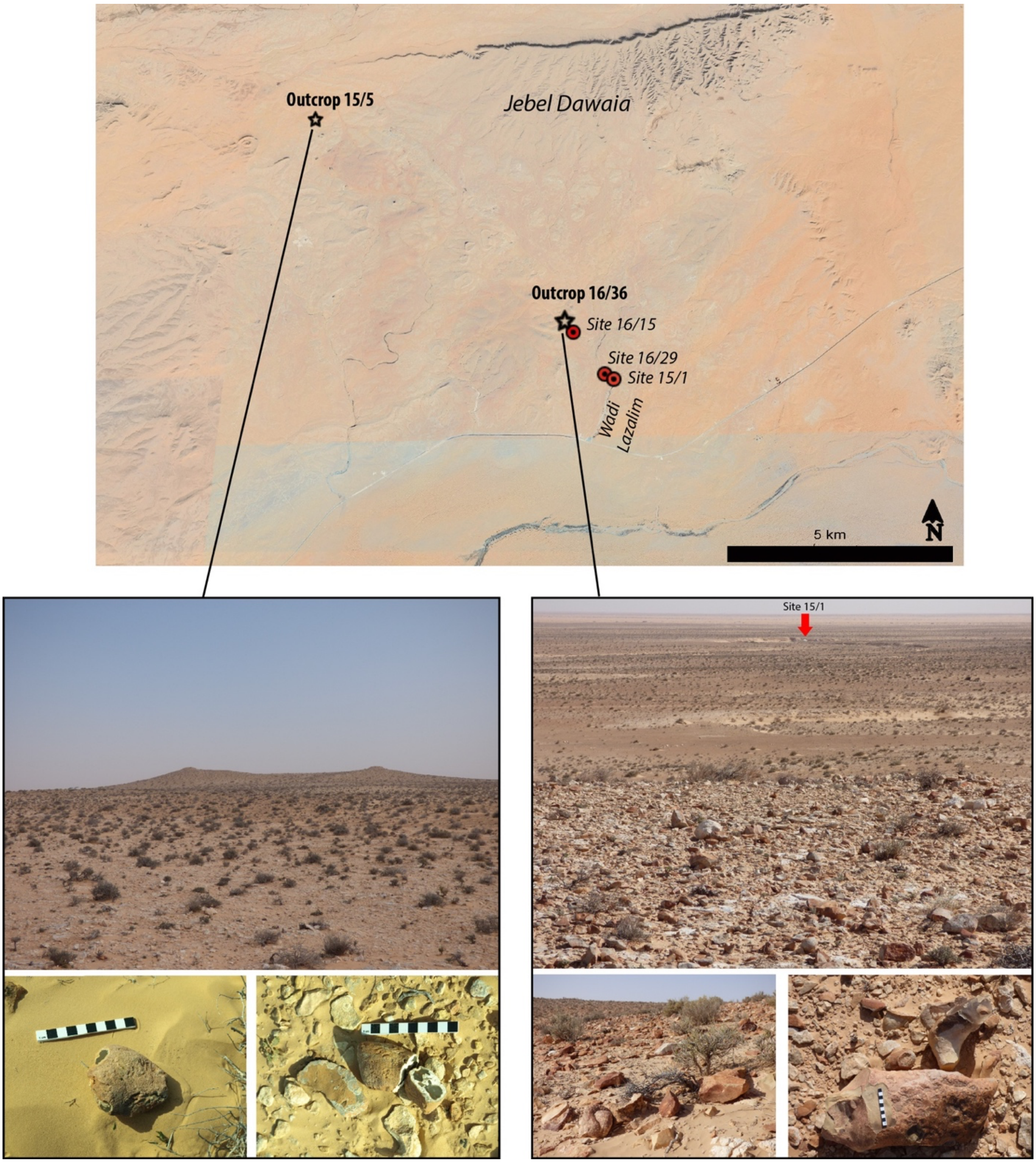


**Fig. SI 7:** Localization of the flint outcrop (16/36), in the very close surroundings of the sites, supposed to be one of the main raw material sources for the artifacts recovered, and of an outcrop of very fine grained black and grey flint nodules (outcrop 15/5) localized some 10 km NW of the sites. (base image Google Earth; map created using QGIS 3.20.2 https://qgis.org/it/site/; scale bars beside flint blocks are 10 cm).

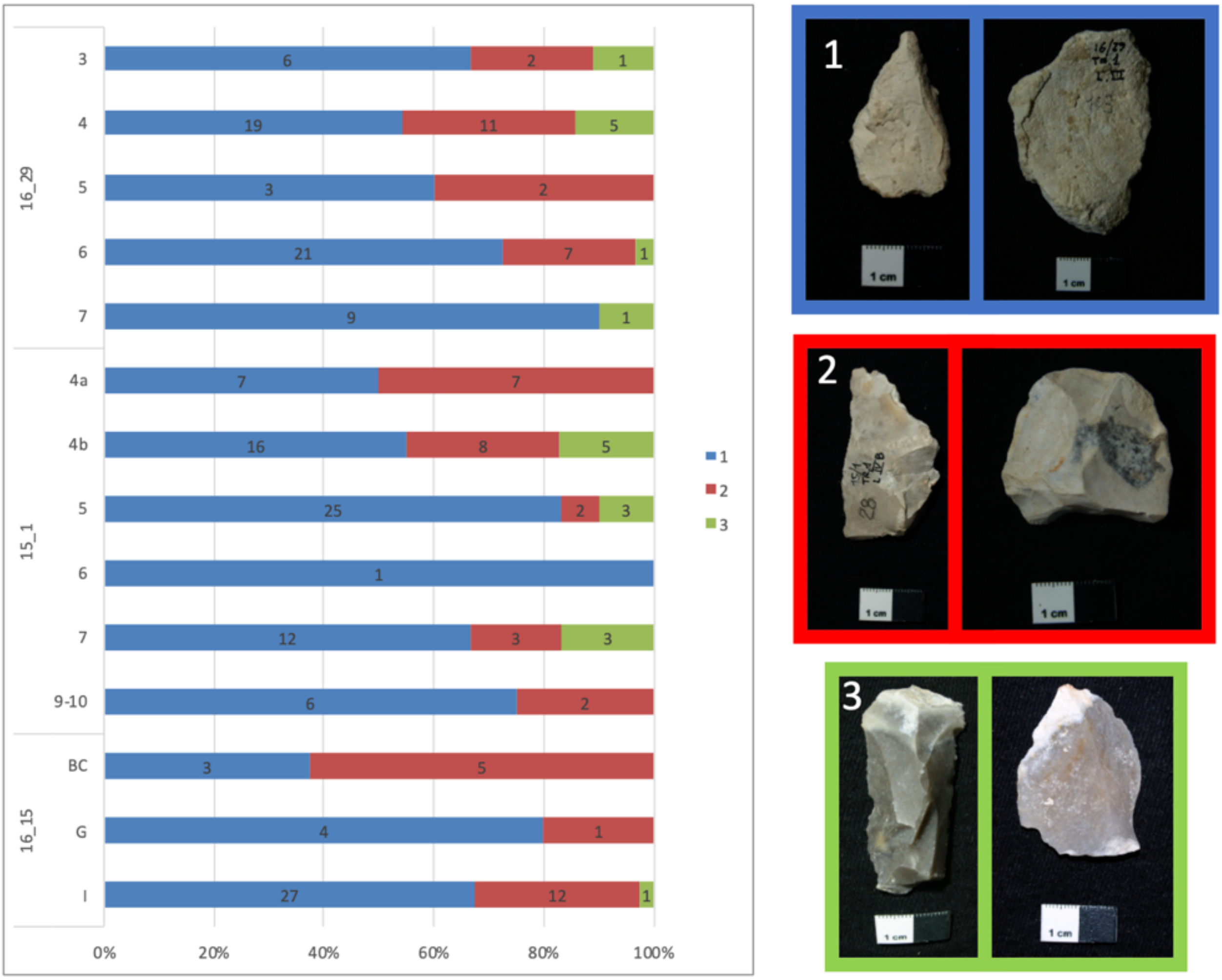


**Fig. SI 8:** Number and incidence of artefacts, per site and layer, classified into three groups of degree of weathering, as macroscopically observed on a sample (n = 241) from 2016 excavation. Groups are briefly defined as: 1, medium grained flint, white patina and invasive surface alterations, moderately affected by mechanical alterations. 2, fine grained flint, whitish or greyish patina, moderately affected by mechanical alterations. 3, very fine grain flint, moderate to no presence of patina, moderately or very little affected by mechanical alterations. Some examples of representative artefacts of each group are provided to the right side of the figure.

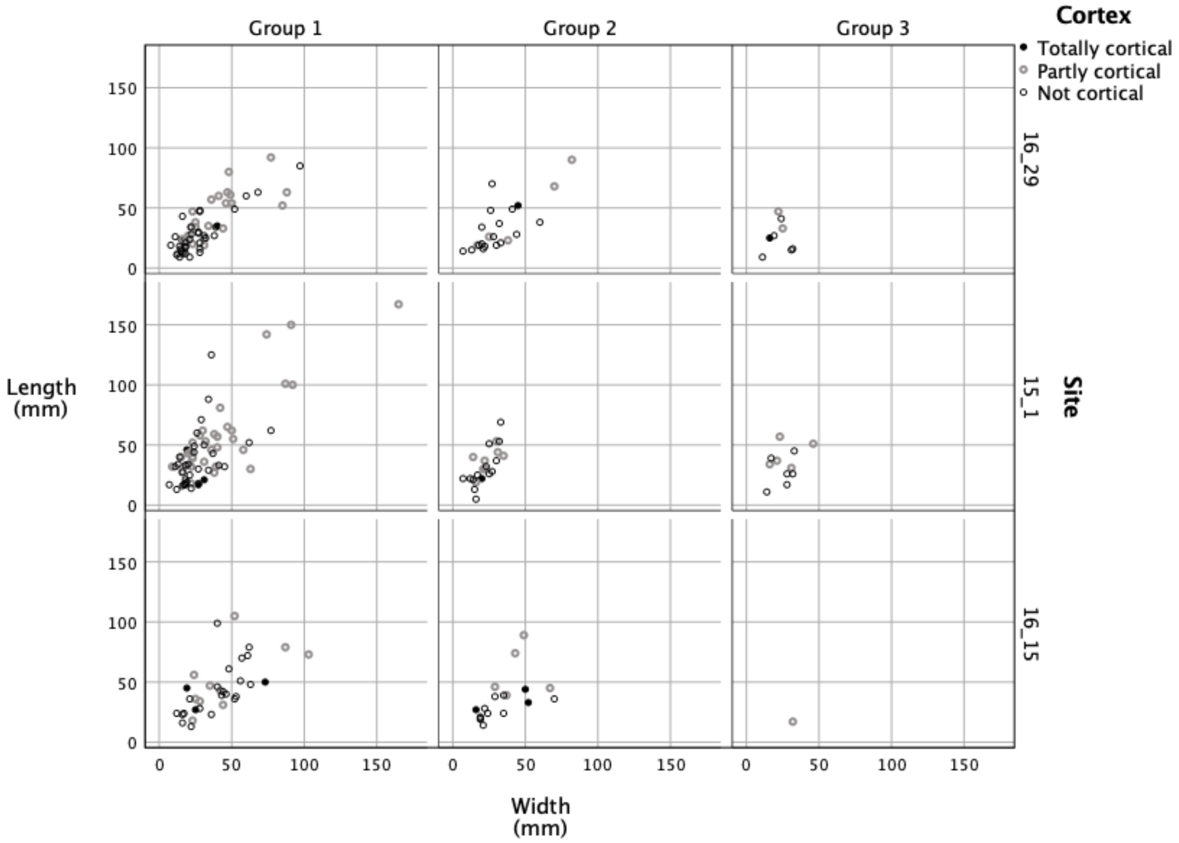


**Fig. SI 9:** Scatter plot of length and width values (in mm) of the artefacts' sample (n = 241) described in Fig. SI 8 distinguished according to Group, Site and presence of cortex.

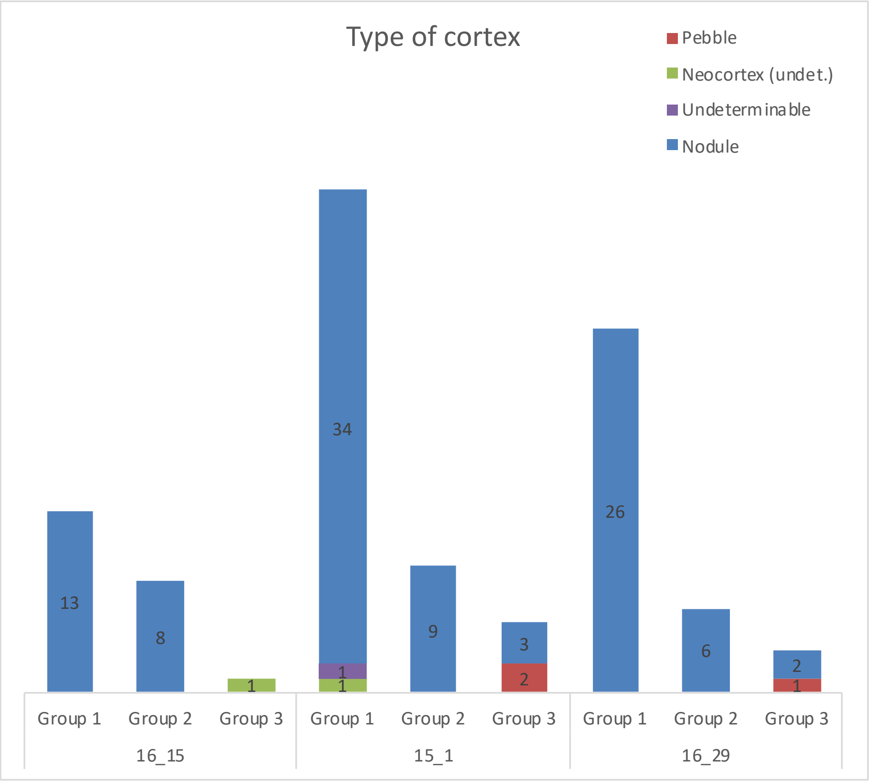


**Fig. SI 10:** Type of cortex recognized on cortical artefacts (sample n = 241).

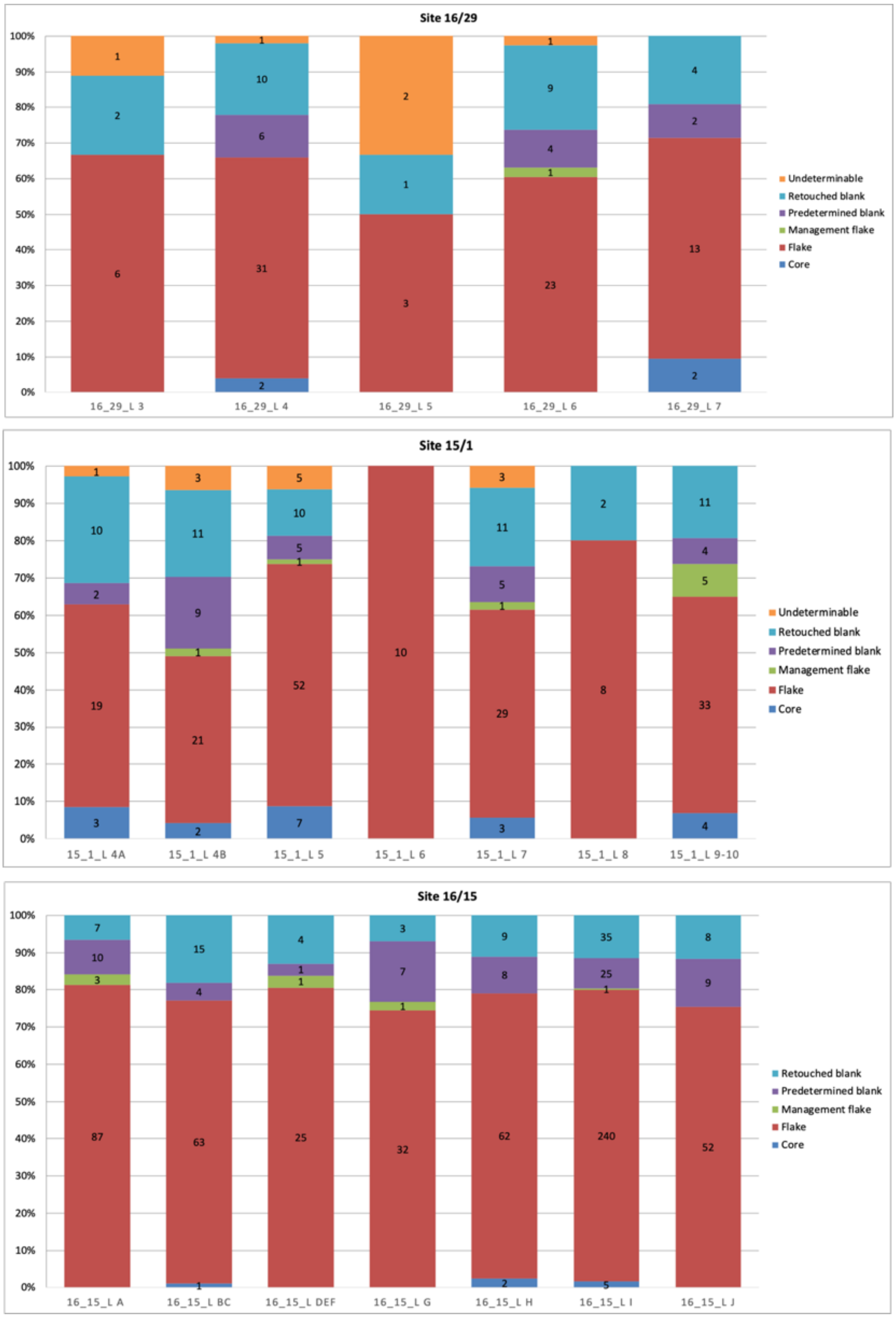


**Fig. SI 11:** Number and incidence of artefacts’ macro-classes per site and layer

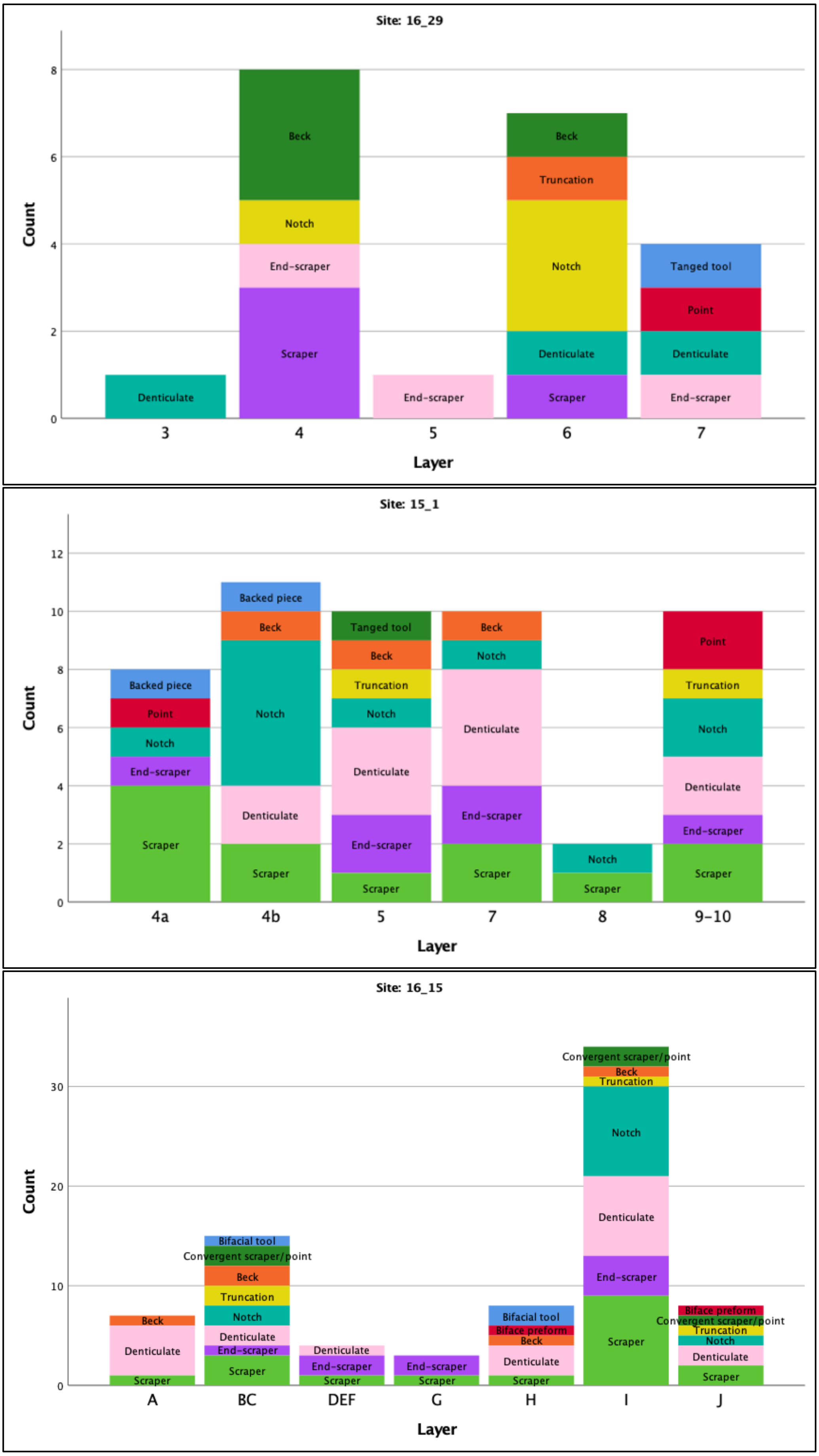


**Fig. SI 12:** Tool type count per site and layer

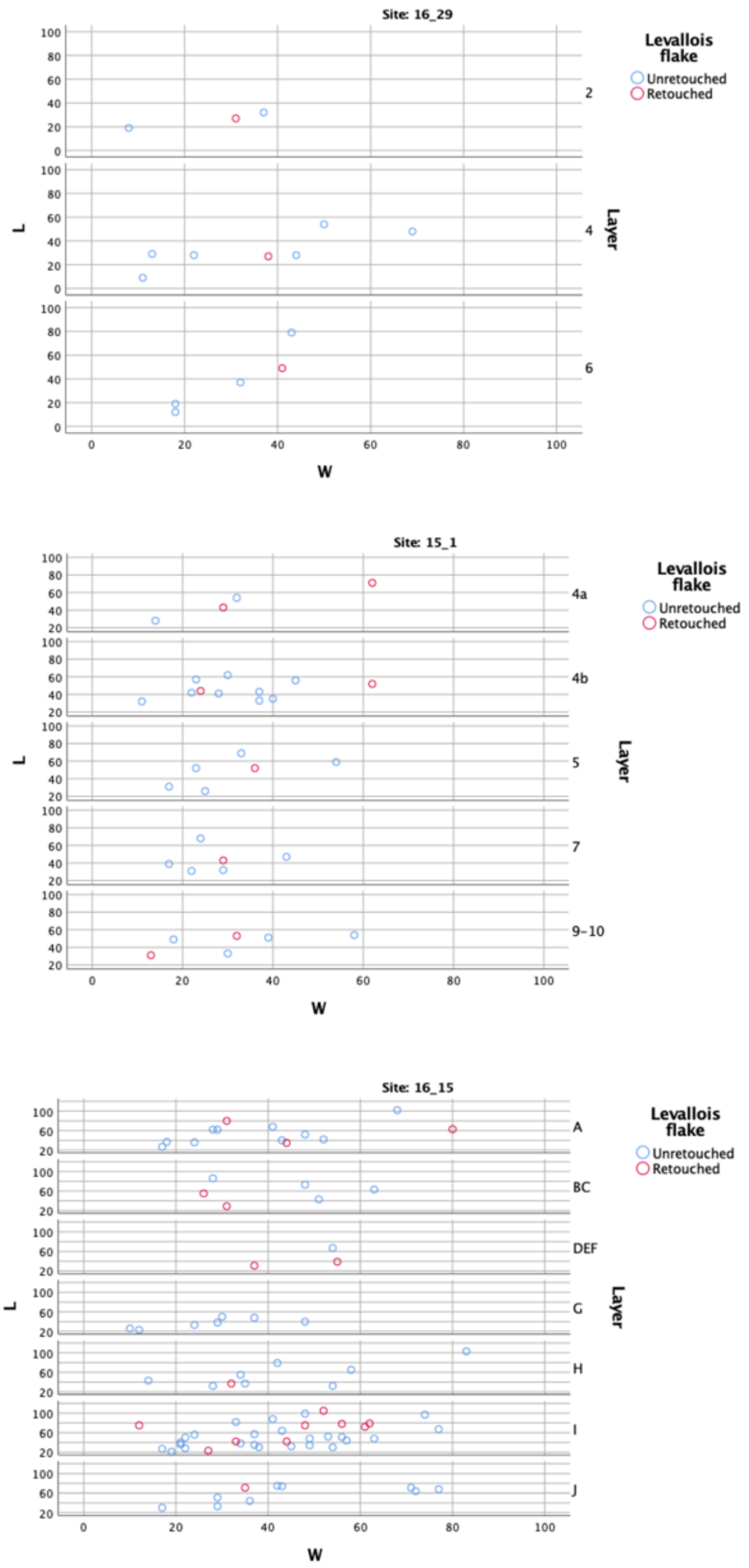


**Fig. SI 13:** Length (L) and width (W) values (in mm) of retouched and unretouched Levallois flakes per site and layer

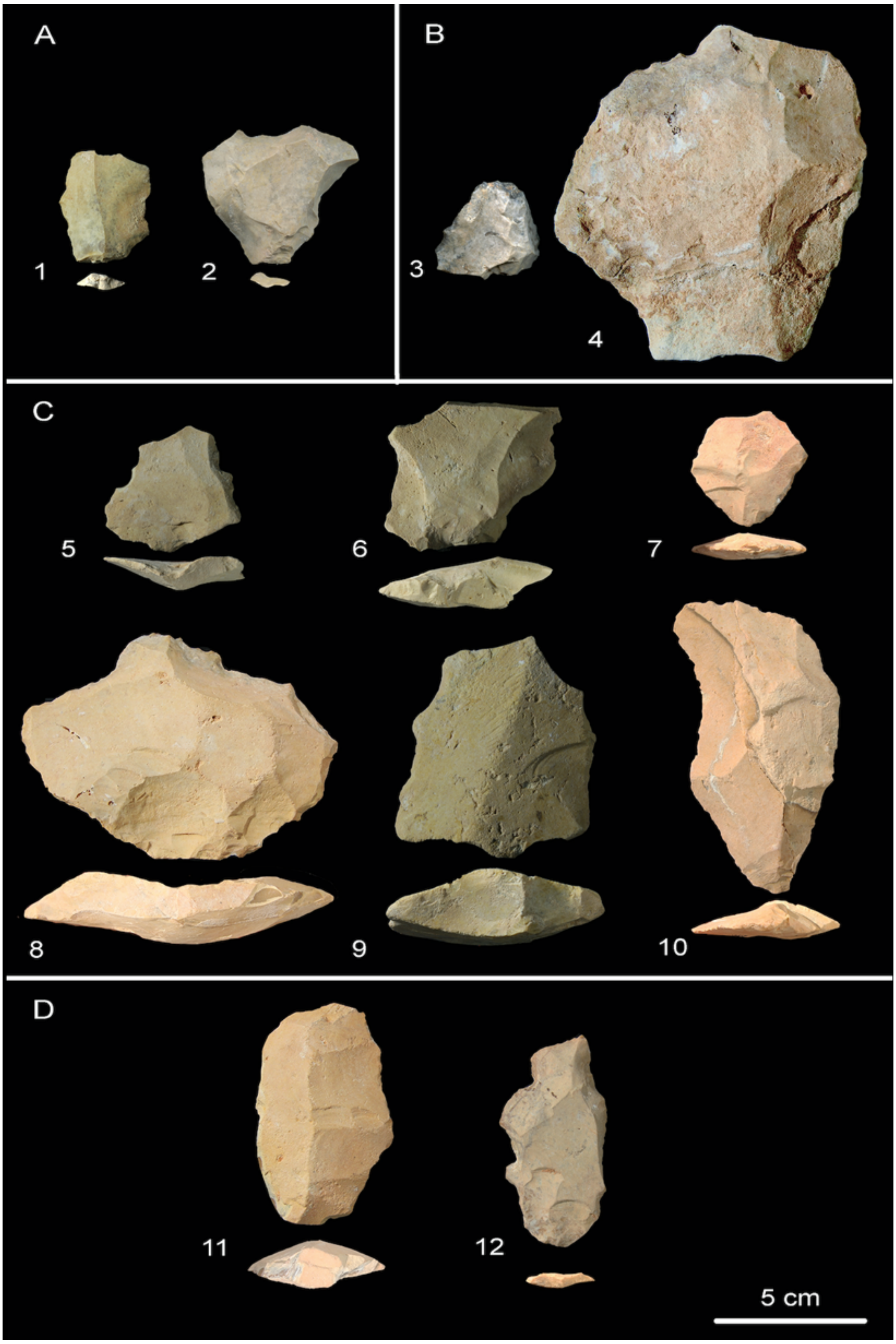


**Fig. SI 14:** Retouched and unretouched Levallois artefacts from site 16/15. 1, 2, 4 (distal fragment), 6, 7, 10, 11: unretouched Levallois flake; 3: scraper (distal fragment); 5, 8, 9, 12: notched piece. A: layer G; B: layer H; C: Layer I; D: Layer J.

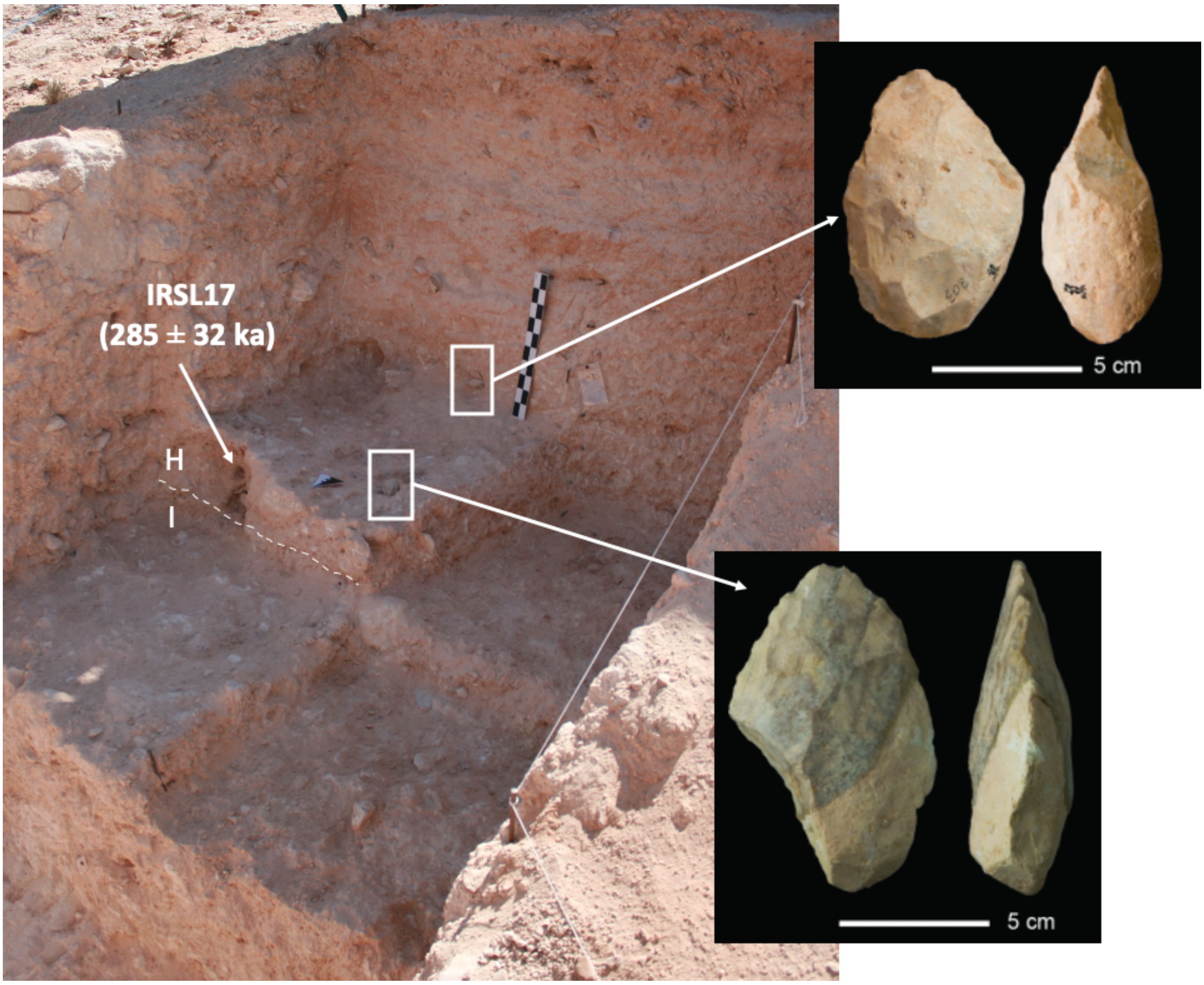


**Fig. SI 15:** Site 16/15 in course of excavation (2017). The position of sample IRSL 17 is indicated. Dashed line designates the limit between layer H and layer I. The rectangles highlight the position of the bifacial tools (knives), still in place, depicted in main text Fig. 1 n. 4 (top) and Fig.1 n. 5 (bottom). Scale bar leaning on south profile is 50 cm.
